# circRanGAP1/miR-27b-3p/NRAS Axis may promote the progression of hepatocellular Carcinoma

**DOI:** 10.1186/s40164-022-00342-6

**Published:** 2022-11-08

**Authors:** Xia-Hui Lin, Zhi-Yong Liu, Dan-Ying Zhang, Si Zhang, Wen-Qing Tang, Dong-Ping Li, Feng Zhang, Rong-Xin Chen, Shu-Qiang Weng, Ru-Yi Xue, Ling Dong

**Affiliations:** 1 Department of Gastroenterology and Hepatology, Zhongshan Hospital, Fudan University, Shanghai, 200032 China; 2 Shanghai Institute of Liver Disease, Shanghai, 200032 China; 3 Key Laboratory of Glycoconjugate Research Ministry of Public Health, Department of Biochemistry and Molecular Biology, School of Basic Medical Sciences, Fudan University, Shanghai, 200032 China; 4 Liver Cancer Institute, Zhongshan Hospital, Fudan University and Key Laboratory of Carcinogenesis and Cancer Invasion, Ministry of Education, Shanghai, 200032 China

**Keywords:** Circular RNA, Progression, Hepatocellular carcinoma, Immune-related genes, Tumor-associated macrophage

## Abstract

**Background:**

Though **c**ircular RNAs (circRNAs) are the key regulators in tumor carcinogenesis, they remain largely unexplored in hepatocellular carcinoma (HCC).

**Methods:**

The expression of RanGAP1-derived circRNAs (circ_0063531, circ_0063534, circ_0063513, circ_0063518, circ_0063507, circ_0063723) were evaluated in eight paired HCC and normal tissues, and the correlation between circRanGAP1 (circ_0063531) expression and clinicopathological characteristics in 40 HCC patients was determined. The association between miR-27b-3p and circRanGAP1 or NRAS was predicted using bioinformatics analysis. The expression of circRanGAP1, miR-27b-3p, and NRAS were detected by quantitative real-time polymerase chain reaction (qRT-PCR). The potential oncogenic role of circ-RanGAP1 was assessed using CCK-8, colony formation, transwell assays in vitro, subcutaneous tumor mouse model, vein tail metastatic model, and orthotopically implanted intrahepatic HCC model in vivo. Luciferase reporter and RNA immunoprecipitation (RIP) assays were used to explore the binding site between miR-27b-3p and circ-RanGAP1 or NRAS. Protein expression was detected using western blotting. The localization of miR-27b-3p and circ-RanGAP1 was investigated using fluorescence in situ hybridization (FISH). The level of immune infiltration was assessed by bioinformatics analysis, flow cytometry, and orthotopically implanted intrahepatic HCC models.

**Results:**

Here, we found elevated circRanGAP1 in the cells and clinical tissues of patients with HCC. Increased circRanGAP1 levels are associated with enlarged tumors and the advanced stage of TNM. CircRanGAP1 promotes the growth, migration, and HCC cell invasion, concurrently with the growth and metastasis of tumors in-vivo. Moreover, circRanGAP1 is mainly located inside the cytoplasm. Mechanistically, circRanGAP1 as an oncogene promotes HCC progression by miR-27b-3p/NRAS/ERK axis, furthermore, affects the infiltration level of tumor-associated macrophages probably by sponging miR-27b-3p. Immune infiltration analysis shows that NRAS is positively correlated with the levels of CD68+ tumor-associated macrophages in HCC samples and that NRAS and CD68 are related to the poor outcome of HCC.

**Conclusion:**

These results reveal that circRanGAP1 is a HCC oncogene that function by the miR-27b-3p/NRAS/ERK axis and regulates the infiltration levels of tumor-associated macrophages by sponging miR-27b-3p. Therefore, circRANGAP1/ NRAS axis may be an important potential treatment target against HCC.

**Supplementary Information:**

The online version contains supplementary material available at 10.1186/s40164-022-00342-6.

## Background

Globally, cancer remains to be the 2nd most common cause of mortality although the death rate has declined significantly. Where liver cancer is ranked 5th most frequent cause of death by cancer in men (6%) and 7th in women (4%) in America [1]. In China, liver cancer ranks fourth among all types of cancer (9.2%) and is the 3rd leading cause of death by cancer (12.9%) [2]. Hepatocellular carcinoma (HCC) is associated with 75–85% of cases of primary liver cancer and is mainly induced by hepatitis viruses (B or C), food products with aflatoxin, and alcohol [3]. Generally, patients with an early diagnosis of HCC receive multiple treatments (e.g., drugs, surgery, and liver transplantation), and therefore may have a better prognosis [4, 5]. However, in light of drug resistance and diagnosis at an advanced stage, many HCC patients still suffer from poor prognoses even after surgical resection [6, 7]. Hence, for the development of future HCC treatments and a deep understanding of HCC pathogenesis, it is essential to identify novel biomarkers and therapeutic targets.

CircRNAs differ from conventional linear RNA in that circRNAs are covalently closed loops without downstream 3’- and upstream 5’- splice sites [8, 9]. It was reported that circRNAs are ubiquitous in mammals and can regulate gene function by binding to microRNAs or other molecules [10]. Furthermore, dysregulation of circRNAs can result in various diseases, such as vascular disease, neurological disorders, and diabetes [11]. Moreover, increasing evidence indicates that circRNAs contribute to the development of various cancers, including gastric cancer, ovarian cancer, and HCC [12–14]. Many circRNAs have been identified as oncogenes in HCC [15–17]. However, there remains an urgent need to explore additional circRNAs for HCC treatment.

Several studies revealed that RanGAP1 plays specific roles in multiple tumors and can affect various physiological processes. Such as, it was reported that one of the important targets for the treatment of brain tumors may be RanGAP1 [18]. Downregulation of RanGAP1 can enhance imatinib efficacy in chronic myeloid leukemia cells [19]. These results revealed that RanGAP1 may participate in tumor progression and has potential value in cancer research. Since RanGAP1 was previously reported to be involved in the development of cancer, we question whether RanGAP1-derived circRNAs might also be involved in cancer progression. Meanwhile, it has been reported that circRanGAP1 can promote gastric cancer progression [12]. Therefore, we hypothesized that RanGAP1-derived circRNAs may be related to the HCC progression. Nevertheless, few studies on circRanGAP1 have been reported, especially in HCC.

To evaluate the role played by circRanGAP1 in HCC, we utilized a variety of technologies (PCR, a luciferase reporter assay, western blotting, etc.) and observed that circRanGAP1 enhances the metastasis, proliferation, and invasion of HCC cells. KEGG analysis also suggested that the MAPK signaling pathway may be a potential downstream pathway for circRanGAP1. Furthermore, previous studies have identified NRAS as an immune-related gene that induces tumorigenesis by altering the immune microenvironment [20–22]. Therefore, we hypothesized that circRANGAP1 accelerated the HCC progression by regulating miR-27b-3p/NRAS axis, activating the downstream MAPK signaling pathway, and affecting the infiltration levels of tumor-associated macrophages.

## Materials and Methods

### Clinical samples and cell lines

Human liver cells (L02), HCC cells (PLC/PRF/5, Hep3B, and HepG2) (Cell Bank of the Chinese Academy of Sciences, Shanghai, China), MHCC97L, MHCC97H, HCCLM3 (Liver Cancer Institute, Fudan University, Shanghai, China), and Huh7 (Japanese Cancer Research Resources Bank) were cultured in Dulbecco’s modified Eagle’s medium (DMEM) (Gibco) which was augmented with fetal bovine serum 10% (Gibco) and penicillin-streptomycin 1% (Invitrogen). Cells were cultured in a thermostatic incubator at 37 °C temperature, humidified atmosphere with 95% air and CO_2_ 5%_._

Ethical approval was obtained from the Research Ethics Committee of Zhongshan Hospital of Fudan University (Shanghai, China), and each patient was asked for written informed consent. 40 HCCs and 40 normal tissues from patients, who received curative resection treatment at the Liver Cancer Institute of Zhongshan Hospital of Fudan University (Shanghai, China) in 2015, were obtained. HCC pathological diagnosis was confirmed, and the medical records were checked for further clinicopathological information. Eight pairs of samples were chosen arbitrarily that used for initial gene screening for detecting the level of RanGAP1-derived circRNAs (circ_0063531, circ_0063534, circ_0063513, circ_0063518, circ_0063507, circ_0063723). And the expression of circRanGAP1 (circ_0063513) in these 40 HCC and matched non-tumor tissues were further analyzed.

### Data processing

TCGA dataset (including 50 normal and 374 tumor samples) was collected from The Cancer Genome Atlas (TCGA) project (https://portal.gdc.cancer.gov/). Gene expression in fragments per kilobase per million (FPKM) format was converted to transcript per million reads (TPM) and then quantile-normalized.

### Identification of miRNAs’ and circRanGAP1’s targets

The potential miRNA targets of circRanGAP1 were predicted utilizing the StarBase (https://starbase.sysu.edu.cn/), TarBase, and RegRNA databases. And the miRNAs that targeted the 3-′UTR of NRAS were obtained from the TargetScan_7.1 (http://www.targetscan.org/vert_71/), Starbase, miRDB (http://mirdb.org/), and miRTarbase (http://mirtarbase.mbc.nctu.edu.tw/index.html) database. And the intersecting miRNAs from these databases were included for further study.

### Plasmid, shRNA, miRNA mimic and inhibitor transfection

The miR-27b-3p mimic, inhibitor, and negative control were obtained from RiboBio (Guangzhou, China). miR-27b-3p mimics, inhibitor, negative control, and plasmids transfection was performed using Lipofectamine 3000 (RiboBio, Guangzhou, China), by following the instructions of the manufacturer.

### Quantitative real-time PCR

Total RNA including cells/tissues miRNA was extracted by utilizing TRIzol Reagent (Invitrogen), and synthesis of cDNA from RNA was done through Reverse Transcription Kit (Takara). Subsequently, cDNA was amplified using the Maxinma SYBR Green qPCR Master Mix (Thermo Scientific). We quantified target genes by the 2^−ΔΔCt^ method and normalization was done by glyceraldehyde-3-phosphate dehydrogenase (GAPDH). miR-27b-3p and U6 Primers were also purchased from RiboBio (Guangzhou, China). The sequences were then covered by the patents. miRNA expression was normalized to that of the internal control, U6, using the 2−ΔΔCT method. For evaluating PCR product specificity Melting curve analysis was performed. Additional file 1: Table S2 enlists the primers used.

### Western blot analysis

Radioimmunoprecipitation assay was applied for the cells/tissue proteins extraction using cell lysis buffer (RIPA) with cocktail protease inhibitor and for mitochondrial protein extraction, a mitochondrial isolation kit (Beyotime Biotechnology) was used, in accordance with the manufacturer's directions. Proteins quantification was done by BCA kit, and then 12% gel was used for SDS-PAGE separation of proteins. Proteins were then transferred to PVDF membranes (0.45 μM) (Millipore, USA). The membrane was then blocked with skimmed milk before primary antibodies incubation overnight at 4 °C, followed by corresponding HRP-conjugated secondary antibody (PeproTech) incubation. Bands were visualized via chemiluminescence. The protein expression intensity was calculated, using Image J software. The antibodies are enlisted in Additional file 1: Table S3.

### CCK-8, clone formation, transwell migration, and invasion assays

The 96 well plate was seeded with 2000 transfected cells/well (Biosciences, USA) at 0, 24, 48, 72, and 96 h. The 10 µL CCK-8 reagent per well was incorporated at each time point following incubation at the temperature of 37 °C for 1–2 h. Wound-healing assay to evaluate cell migration function was performed. To produce a linear wound cell monolayer was disrupted mechanically with the help of a sterile pipette tip (200 μl). The cells’ migratory distance was observed under the microscope. For the transwell assay, 5 × 10^4^ cells in 200 µL serum-free DMEM were incorporated in the upper chamber ((8 µm pore size; BD Falcon, San Jose, CA)) of the well with DMEM with fetal bovine serum 10%. For the invasion assay, in 24-well Transwell plates (8 μm pore size, Corning, NY, USA) suspended cells (1 × 10^5^) were seeded in the serum-free medium into the 1 µg/µL Matrigel-coated upper chamber (BD Biosciences, USA), and in the lower chamber DMEM (600 μl) +FBS 10% was added following incubation at the temperature of 37 °C and 5% CO_2_. The migrating and invading cells on the outer side of the upper chamber membrane were then fixed using 4% paraformaldehyde before staining with crystal violet and counted under a light microscope (100×magnification).

### Luciferase reporter assay

Co-infection of HCCLM3 and Huh7 cells was achieved with a mixture of 6 ng pRL-CMV Renilla luciferase reporter, 60 ng luciferase, and miR-27b-3p mimic or inhibitor. Cells were incubated for 48 h, and then the firefly and Renilla luciferase activities were determined using a dual-luciferase reporter assay (Promega, Madison, WI, USA).

### RNA immunoprecipitation (RIP) assay

RIP assays were carried out by using a RIP RNA-Binding Protein Immunoprecipitation Kit (BersinBio, Guangzhou, China) by following the protocol suggested by the manufacturer. Additional file 1: Table S3 enlists argonaute 2 (AGO2) and IgG antibodies.

### Knockdown or overexpression of circRanGAP1

HCC cells with circRanGAP1 knockdown/overexpression were constructed by utilizing lentivirus. Lentiviral vectors that encoded shRNA targeting circRanGAP1, overexpression of circRanGAP1 or NC shRNA, and mock were obtained from GENECHEM Gene Company (Shanghai, China). Lentivirus particles were used for transfecting cells with (multiplicity of infection =10) and then selected in a culture medium with 2 μg/ml puromycin. The gene silencing efficiency was assessed using quantitative real-time PCR. The target shRNA sequences are enlisted in Additional file 1: Table S4.

### RNA fluorescence in situ hybridization (FISH)

Overnight hybridization was achieved with circRanGAP1 (5’-CY3-ACTCCAGTGGCAGCGTGAAGCTGCCACACG-CY3-3’) and miR-27b-3p (5’-FAM-gCAGAACTTAGCCACTGTGAa-FAM-3) probes (Additional file 1: Table S5). After prehybridization, circRanGAP1and miR-27b-3p probes were hybridized in a hybridization buffer, and DAPI was used for nuclei staining (Yeasen, Shanghai, China), then circRanGAP1and miR-27b-3p were observed under a confocal microscope. The circRanGAP1 and miR-27b-3p probes were synthesized by Bersin Bio (Guangzhou, China).

### RNase R treatment

Total RNA (2µg) with and without 3 U/µg RNase R was incubated at 37 °C temperature for 20 min. A RNeasy MinElute Cleanup Kit (Qiagen) was used for final RNA purification.

### Actinomycin D assay

Huh7 and HCCLM3 cells were treated with actinomycin D (2 µg/mL) (Sigma) for 4, 8, 12, or 24 h to block transcription. Quantitative real-time PCR was used for analyzing the stability of circRanGAP1 and RanGAP1.

### Immunohistochemistry (IHC)

As aforementioned, immunohistochemistry was done by utilizing EnVision two-step visualization system (GeneTech, Shanghai, China). Briefly, for de-deparaffinization, xylene was used on tumor tissue sections (5 μm), which were then rehydrated with ethanol dilutions, and then blocked using 3% H_2_O_2_. The sections were then microwaved for antigen retrieval and slides were again blocked, this time using 5% BSA. After blocking it was left for overnight incubation at 4°C in primary antibodies followed by secondary antibodies incubation and lastly, visualization with 3,3-diaminobenzidine (DAB) chromogen. Hematoxylin was used for counterstaining. The slide was observed, and images were taken under the light microscope (Olympus), and the intensity of positive staining was measured. Additional file 1: Table S3 enlists antibodies used in this study.

### Flow cytometric analysis

From fresh tumor tissues, we isolated single cells using collagenase IV, which were then stained with relevant monoclonal antibodies (mAbs) for 30 min at 4 °C temperature. Intracellular proteins were first fixed and then permeabilized using the Fixation/Permeabilization Solution Kit (BD Biosciences), in accordance with the kit’s guidelines. Fluorochrome-conjugated CD3, CD45, CD25, CD8, CD4, FoxP3, NK1.1, B220, MHC-II, CD11c, F480, and CD11b monoclonal antibodies (mAbs) were purchased from BioLegend and eBioscience. After staining cells were washed and then resuspended in PBS (0.1%), azide, and bovine serum albumin. FACS caliber flow cytometer (BD Biosciences, San Jose, CA, USA) and FlowJo software (Tree Star, San Jose, CA) were used for flow cytometry analysis. Additional file 1: Table S3 enlists antibodies used in this study.

### In vivo tumor growth and metastasis model

The approval for using animals during this study was taken from the Committee on Animal Research of Zhongshan Hospital, Fudan University (Shanghai, China). All the experiments were carried out in accordance with the guidelines mapped out by the Shanghai Medical Experimental Animal Care Commission. For the in vivo growth model, twenty BALB/c nude male mice about 4–6 weeks of age with an approximate weight of 18–20 g were acquired from Shanghai SLAC Laboratory Animal Co., Ltd., China, and maintained in a sterile environment. For the in vivo growth model, mice were divided randomly into 4 sets: a suspension of 2 ×107 MHCC97H cells (mock and circRanGAP1) and HCCLM3 cells (NC shRNA and circRanGAP1 shRNA) was given subcutaneously in the right flank of each mouse. On day 25, 20 nude mice in each group were euthanized by anesthesia, and tumor dimensions were measured by applying the following formula: Volume (mm^3^) = [width^2^ (mm^2^) × length (mm)]/2. For the in-vivo metastasis model, the mice were randomly allocated to four groups: mock and circRanGAP1 (MHCC97H), NC shRNA, and circRanGAP1 shRNA (HCCLM3). Five mice were recruited for each group. A lung metastatic tumor model was established by the following protocol: cells (2× 10^7^/ml) were suspended in PBS, and 0.2 ml of this suspension was injected into the tail veins of the mouse. At 6 weeks, twenty-four nude mice in each group were euthanized by anesthesia overdose and visceral organs, including the lungs and livers, were collected. Intrahepatic and pulmonary metastatic foci were examined by pathological identification. For the liver orthotopic xenograft implantation model, flank subcutaneous xenografts were modeled by injecting 1 × 10^7^ cells in 100 μl PBS subcutaneously in C57BL/6 mice (5 weeks old), which were kept by following the 3Rs (reduction, refinement, and replacement) guideline. Subcutaneous tumors, after 2–3 weeks, were removed and their weight and volume were recorded. Furthermore, they were dissected into sections of 1 mm^3 ^and then implanted into the liver parenchyma. For mice in the miR-27b-3p mimic and miR-NC treatment group, 100OD/kg, miR-27b-3p mimic, and miR-NC (dissolved in DEPC) were administered via tail veins eight times (twice a week) before establishing the metastasis model. 6 weeks post experimentation mice were sacrificed, and tumor size was measured by MRI. Lastly, sections of the tumor, liver, and lung were fixed and stained routinely using hematoxylin and eosin (H&E) staining and immunohistochemistry (IHC). Additional file 1: Table S3 enlists antibodies used in this study.

### Statistical analysis

As previously described [23], briefly, the statistical analyses of clinical data were carried out using SPSS software (21.0; SPSS, Inc., Chicago, IL, USA). Using Pearson’s Chi-square test to assess the association between expression of circRanGAP1 and clinicopathological characteristics. OS was represented using Kaplan-Meier analyses (log-rank test). The functional experiments were repeated 3 times, and results were expounded as mean ± SD. Pearson's correlation analysis was utilized for investigating the correlation between two variables. The student’s t-test was done for comparing the variations between the two groups. One-way ANOVA, followed by Tukey’s post hoc analysis, was applied for comparing the differences between multiple groups. And the differences in categorical variables were compared using the chi-square test or Fisher’s exact test, as appropriate. All statistical tests were two-sided, and P < 0.05 indicated a statistical significance difference.

## Results

### Analysis of RanGAP1-derived circRNAs in HCC tissues

**Fig. 1:**
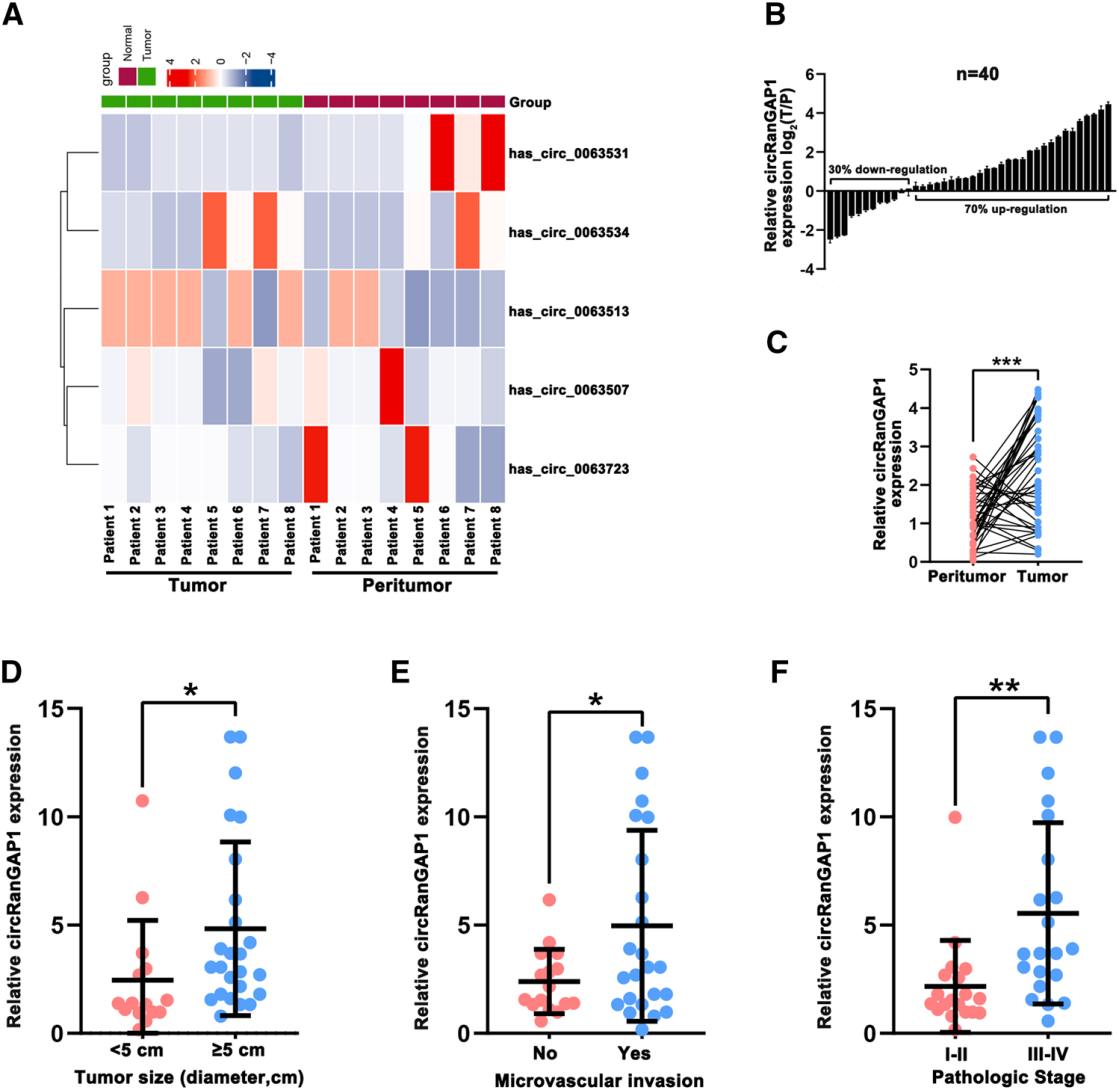
Analysis of RanGAP1-derived circRNAs in HCC tissues. **A**. The heatmap depicts the RanGAP1-derived circRNA expression of 8 paired tissues of HCC and adjacent non-tumor tissues. **B** and **C**. The relative expression of circRanGAP1 in HCC tissues and matched non-tumor tissues of 40 patients. **D**. The circRanGAP1 expression in different tumor size groups (< 5cm and >= 5cm). **E**. The circRanGAP1 expression in groups with or without microvascular invasion. **F**. The circRanGAP1 expression in I-II and III-IV stage groups of 40 patients. The data are represented as the mean ± SD, n=3. *P < 0.05; **P < 0.01; ***P < 0.001; *NS* not significant

In previous investigations, upregulation of RanGAP1 was observed along with its role in the progression of multiple tumors [12, 18, 19]. Herein, the level of RanGAP1-derived circRNAs (circ_0063531, circ_0063534, circ_0063513, circ_0063518, circ_0063507, circ_0063723) was detected in eight paired HCC human samples using quantitative real-time PCR. Circ_0063518 was excepted since its extremely low level, and the results show that circ_0063513 was most significantly upregulated in HCC tissues than that in normal tissues (Fig. 1A). We named circ_0063513 as “circRanGAP1”. And we examined the expression of circRanGAP1 using quantitative real-time PCR in 40 HCC and matched non-tumor tissues, as Fig. 1B, C depict the expression of circRanGAP1 in HCC tissues was higher in 28 (70%), and significantly higher (|log_2_FC| >= 1) in 19 (47.5%) than that in paired normal tissues. The correlation between circRanGAP1 and the clinicopathological manifestations of the 40 HCC specimen was analyzed, and the results (Table. 1) revealed that circRanGAP1 expression level was largely associated with enlarged tumor dimension (P = 0.048) and advanced pathologic stage (P = 0.001). Importantly, we found that high levels of circRanGAP1 (circRanGAP1^high^) in HCC patients had a large-sized tumor (Fig. 1D), more microvascular invasion (Fig. 1E), and an advanced TNM stage (Fig. 1F). Taken together, circRanGAP1 is identified as a potential biomarker in HCC, and enhancement of circRanGAP1 is likely associated with the progression of HCC.

**Table 1:** Correlation between circRanGAP1 and clinicopathological characteristics in 40 HCC patients

Characteristic	circRanGAP1 High	circRanGAP1 Low	p
No. patients	20	20	
Age, n (%)			0.514
< 55	9 (22.5%)	6 (15%)	
>= 55	11 (27.5%)	14 (35%)	
Sex, n (%)			1.000
Female	5 (12.5%)	4 (10%)	
Male	15 (37.5%)	16 (40%)	
Tumor number, n (%)			0.695
>= 2	3 (7.5%)	5 (12.5%)	
1	17 (42.5%)	15 (37.5%)	
Tumor size, n (%)			**0.048**
< 5cm	4 (10%)	11 (27.5%)	
>= 5cm	16 (40%)	9 (22.5%)	
Pathologic stage, n (%)			**0.001**
I-II	4 (10%)	15 (37.5%)	
III-IV	16 (40%)	5 (12.5%)	
Microvascular invasion, n (%)			0.333
No	6 (15%)	10 (25%)	
Yes	14 (35%)	10 (25%)	
S grade, n (%)			0.527
S1-2	8 (20%)	11 (27.5%)	
S3-4	12 (30%)	9 (22.5%)	
HBsAg, n (%)			1.000
Negative	4 (10%)	4 (10%)	
Positive	16 (40%)	16 (40%)	
HCVAb, n (%)			1.000
Negative	1 (2.5%)	2 (5%)	
Positive	19 (47.5%)	18 (45%)	
ALT, U/L, n (%)			0.695
<=75	15 (37.5%)	17 (42.5%)	
>75	5 (12.5%)	3 (7.5%)	
AFP, ng/ml, n (%)			0.320
<=20	5 (12.5%)	9 (22.5%)	
>20	15 (37.5%)	11 (27.5%)	

*AFP* alpha-fetoprotein, *HBsAg* hepatitis B surface antigen, *HCVAb* hepatitis C virus antibody, *ALT* aLanine aminotransferase

*p < 0.05 was regarded as statistically significant, the p-value was calculated using Cox proportional hazards regression. Bold p-value was regarded as statistically significant.

### Characterization of circRanGAP1 in HCC

When RNA sequences of hsa_circ_0063513 and RanGAP1 from circBase were compared, it was revealed that circ_0063513 is looped and contains 4–16 exons of RanGAP1 gene of 2516 bp (on chr22:41641614-41664160) (Additional file 1: Fig S1A). Random hexamer or oligo (dT)_18_ primers were used to explore the potential circular characteristics of circRanGAP1 in HCC. Random hexamer primers comparison revealed that circRanGAP1 levels decreased significantly, whereas linear RanGAP1 levels were not affected (Additional file 1: Fig S1B). This finding demonstrates that circRanGAP1 does not have a poly-A tail. In addition, circRanGAP1 is resistant to RNase R (exoribonuclease for linear RNAs digestion), further confirming that circRanGAP1 has a loop structure (Additional file 1: Fig S1C). Beyond that, actinomycin D was used for inhibiting transcription. The half-life of circRanGAP1 and linear RanGAP1 was measured in Huh7 and HCCLM3 cells. The results indicate that Circular RanGAP1 has a half-life of over 24 h, whereas linear RanGAP1 has an approximate half-life of 4–8 h (Additional file 1: Fig S1D), indicating that circRanGAP1 is highly stable. In addition, cytoplasmic and nuclear RNA analysis (Additional file 1: Fig S1E) and fluorescence in situ hybridization (FISH) (Additional file 1: Fig S1F) against circRanGAP1 show that circRanGAP1 is predominantly located in HCCLM3 and Huh7 cells cytoplasm. Collectively, these studies indicate that circRanGAP1 is a steady, abundant, and circular transcript in HCC.

### CircRanGAP1 facilitates proliferation, migration, and invasion of HCC cells

**Fig. 2:**
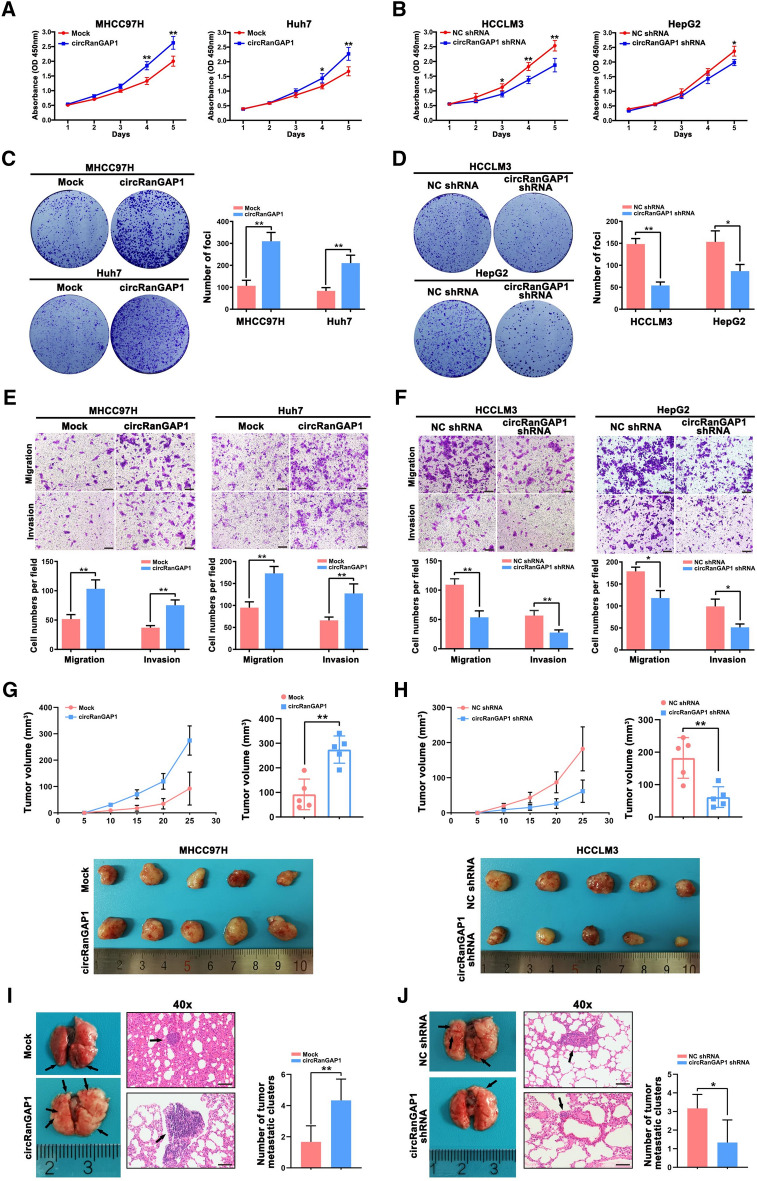
circRanGAP1 facilitates proliferation, migration, and invasion of HCC cells in vitro and in vivo. **A** and** B**. CCK-8 assay was done to detect overexpressed circRanGAP1 MHCC97H and Huh7 cells proliferation and circRanGAP1-downregulated HCCLM3, HepG2, and control cells. **C** and **D**. Colony formation assay was carried out for evaluating proliferation of circRanGAP1-overexpressed MHCC97H and Huh7cells, circRanGAP1-downregulated HCCLM3 and HepG2 cells, and control cells. **E** and** F**. through Transwell migration and invasion assays were performed for the detection of circRanGAP1-overexpressed MHCC97H and Huh7 cells migration and invasion, circRanGAP1-downregulated HCCLM3 and HepG2 cells, and control cells. **G** and** H**. subcutaneous tumor model was used to analyze the growth rate of tumors and tumor size in the circRanGAP1-overexpressed MHCC97H group than in mock group and the circRanGAP1-downregulated HCCLM3 group and not the control group. **I** and **J**. A tail vein metastasis model was prepared to analyze the metastatic ability of cancer cells in the circRanGAP1-overexpressed MHCC97H group than the mock group and the circRanGAP1-downregulated HCCLM3 group than the control group. Data are expounded as the mean ± SD, n=3. *P < 0.05; **P < 0.01; ***P < 0.001; NS, not significant

We analyzed circRanGAP1 levels in HCC cells using quantitative real-time PCR and discovered that circRanGAP1 is generally higher in six HCC cell lines (MHCC97H, Huh7, MHCC97L, PLC, HCCLM3, and HepG2) than that in L02 cells, a normal hepatic cell line. HCCLM3 and HepG2 cells have a higher circRanGAP1 expression, whereas MHCC97H and Huh7 cells have lower circRanGAP1 levels (Additional file 1: Fig S2A). Therefore, MHCC97H and Huh7 cells were chosen for circRanGAP1 overexpression and HCCLM3 and HepG2 cells were selected for circRanGAP1 knockdown. circRanGAP1 overexpressing plasmid was constructed and circRanGAP1 overexpression efficiency was tested in MHCC97H and Huh7 cells (Additional file 1: Fig S2B). Furthermore, a shRNA targeting the circRanGAP1 back-splice junction site was specifically designed to downregulate the relevant expression, and the knockdown efficiency was evaluated by quantitative real-time PCR (Additional file 1: Fig S2C). To confirm that circRanGAP1 shRNA does not affect the other RanGAP1 splicing products, for the detection of RanGAP1 mRNA expression quantitative real-time PCR was carried out. RanGAP1 mRNA expression did not any significant change post-transfection with circRanGAP1 shRNA (Additional file 1: Fig S2D), confirming the specificity of circRanGAP1 knockdown in HCCLM3 and HepG2 cells. Both CCK-8 and colony formation assays uncovered that circRanGAP1 overexpression markedly accelerated HCCLM3 and HepG2 cell growth (Fig 2A, C), while downregulation of circRanGAP1 exerted the opposite effect (Fig 2B, D). Transwell assays also reveal that overexpressed circRanGAP1 promotes HCC cell migration and invasion (Fig 2E), and that knockdown of circRanGAP1 exhibits the opposite effect (Fig 2F). Subsequently, the circRanGAP1 effects on HCC cell development and metastasis were then further investigated using a subcutaneous tumor model and a tail vein metastasis model in nude mice. In the subcutaneous tumor model, there was a significant increase in tumor growth rate in circRanGAP1 overexpressing group than in the mock group (Fig 2G). Similarly, the tumor size was enhanced remarkably in the overexpressing circRanGAP1 group than in the control group (Fig 2G). In contrast, the growth rate and size of tumors were reduced after the knockdown of circRanGAP1 (Fig. 2H). In the tail vein metastasis model, the number of metastatic nodules in the pulmonary tissues rose in the circRanGAP1 overexpressing MHCC97H cells (Fig.2I); but decreased in the circRanGAP1 downregulated HCCM3 cells (Fig 2J). Overall, the results demonstrated that circRanGAP1 functions as an oncogenic circRNA to promote the invasion, maturation, and migration of HCC cells in both in-vitro and in-vivo conditions.

### CircRanGAP1 acts as a sponge of miR-27b-3p in HCC cells

**Fig. 3:**
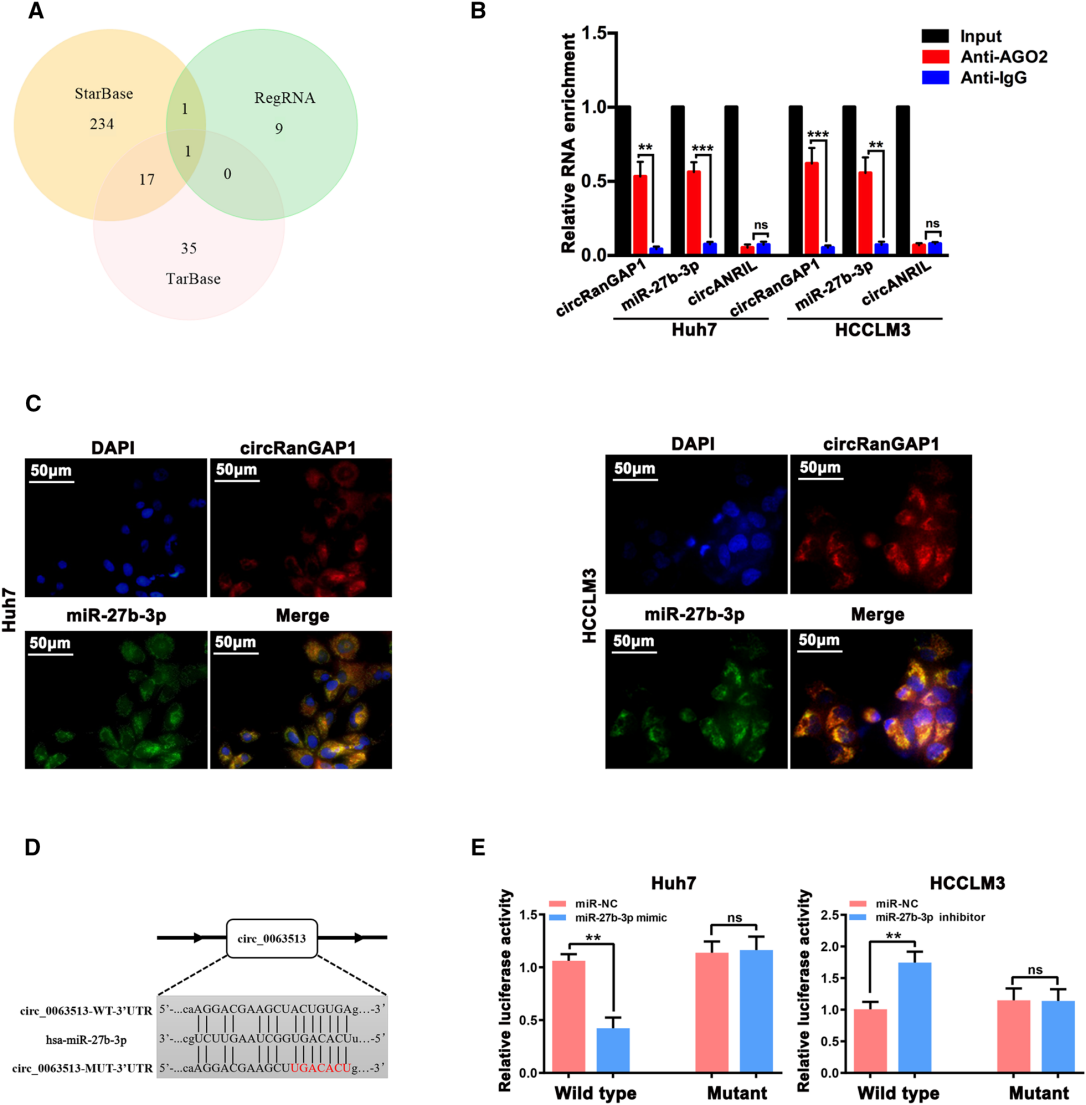
circRanGAP1 acted as a sponge of miR-27b-3p in HCC cells. **A**. Venn diagram exhibiting the overlap of circRanGAP1 target miRNAs obtained from Starbase, Tarbase, and RegRNA databases. **B**. AGO2 antibody was used for performing RIP experiments on extracts from Huh7 and HCCLM3 cells. **C**. circRanGAP1 and miR-27b-3p were evaluated by RNA FISH in Huh7 and HCCLM3 cells. Nuclei were stained with DAPI. Scale bar, 10μm. **D**. Putative binding site between miR-27b-3p and circRanGAP1 was predicted via StarBase v3.0. **E**. The luciferase activity of wildtype-circRanGAP1 or mutant-circRanGAP1 after miR-27b-3p mimic co-transfection in Huh7 cells or inhibitor in HCCLM3 cells. The data are represented as the mean ± SD, n=3. *P < 0.05; **P < 0.01; ***P < 0.001; *NS* not significant

In the cytoplasm, circRNAs may act as miRNA sponges [9, 24, 25]. Here, we explored whether circRanGAP1 could interact with certain miRNAs in HCC. Firstly, an online database: circRNA Interactome (https://circinteractome.nia.nih.gov/index.html) suggests enhanced Ago2, circRanGAP1 region occupancy in the (Additional file 1: Fig S2A), secondly, we demonstrated that circRanGAP1was predominantly located in the HCC cells cytoplasm through nuclear/cytosol fractionation and fluorescence in situ hybridization (FISH) assays. Thirdly, online databases including StarBase v2.0, RegRNA, and TarBase were used to predict the potential targets of RanGAP1. And all the potential targets were listed in Additional file 1: Table S5. We identified that circRanGAP1 specifically targeted only miR-27b-3p (Fig. 3A) and validated this result with an RNA immunoprecipitation assay (RIP) in Huh7 and HCCLM3 cells using an anti-argonaute2 (AGO2) antibody. The results revealed significant expression of circRanGAP1 and miR-27b-3p and not circANRIL (circular RNA lack AGO2 binding), after AGO2 antibody binding experiments (Fig. 3B), suggesting that circRanGAP1 may co-exist with miR-27b-3p by binding to each other in HCC cells. FISH assays further demonstrated that both circRanGAP1 and miR-27b-3p are predominantly cytoplasmic content of Huh7 and HCCLM3 cells. Notably, they are also co-localized in the cytoplasm (Fig. 3C). Furthermore, vectors of luciferase reporter (comprising miR-27b-3p targets mutant (Mut) or wild-type (WT) sequence) were co-transfected with the miR-27b-3p inhibitor or mimic into both the cell types. The effect of the WT circRanGAP1 vector was alleviated by the miR-27b-3p mimic in Huh7 cells but enhanced by the miR-27b-3p inhibitor in HCCLM3 cells. However, no effect was observed in luciferase activity produced by the miR-27b-3p mimic or inhibitor in mutant vector-transfected Huh7 and HCCLM3 cells (Fig. 3D, E). Interestingly, the level of circRanGAP1 decreased and increased after up-regulation and down-regulation of miR-27b-3p expression in Huh7 and HCCLM3 HCC cells, respectively. Meanwhile, miR-27b-3p levels decreased and increased after the overexpression and knockdown of circRanGAP1 in Huh7 and HCCLM3 cells, respectively (Additional file 1: Fig S3B, C). These findings suggest that circRanGAP1 acts as a sponge for circRanGAP1 and miR-27b-3p, where miR-27b-3p may modulate the expression of each other.

### miR-27b-3p as a tumor suppressor reverses circRanGAP1’s effects on HCC cells

**Fig. 4:**
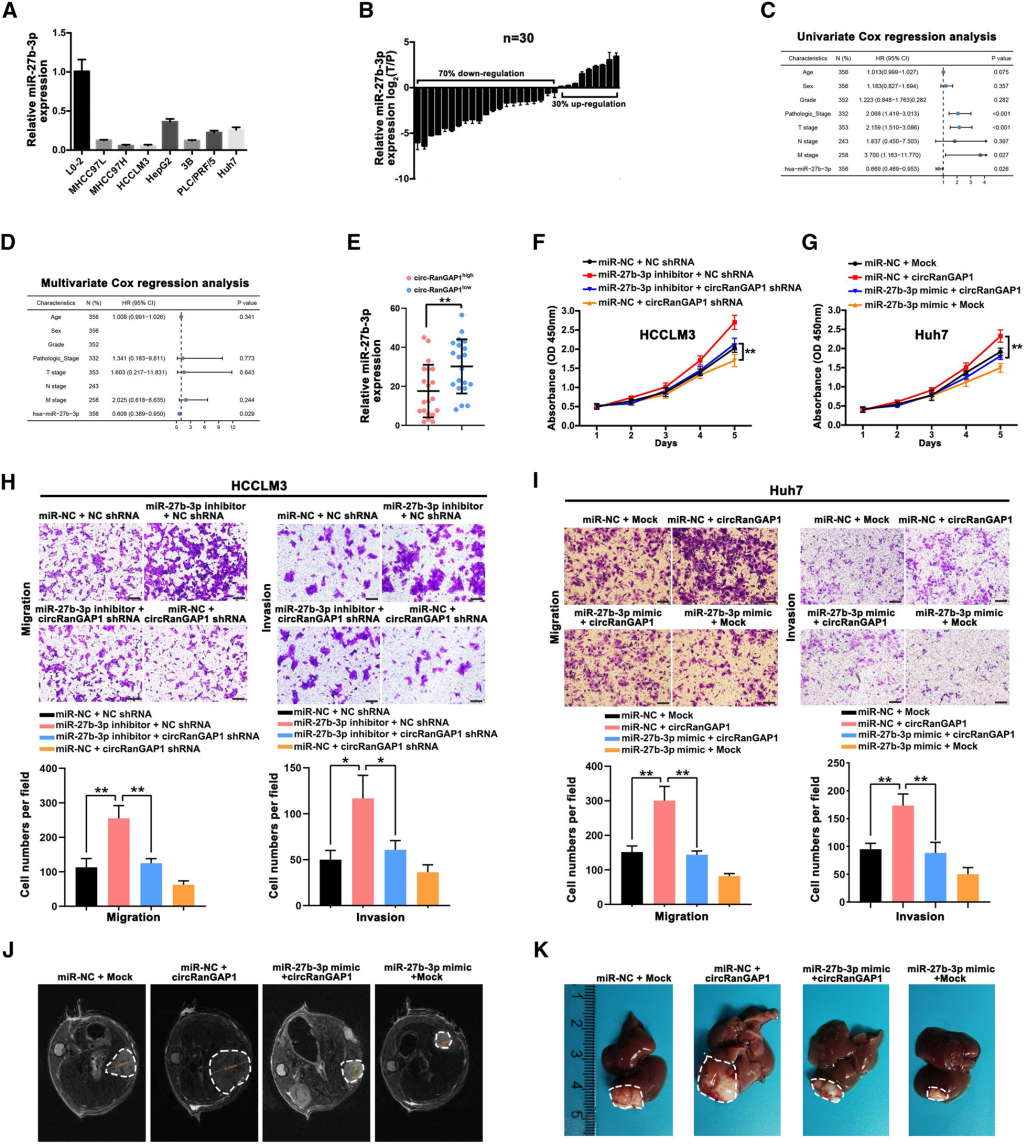
miR-27b-3p, as a tumor suppressor, reverses circRanGAP1’s effects on HCC. **A**. The miR-27b-3p expression in seven HCC and normal liver cell L02. **B**. The relative miR-27b-3p expression in HCC and comparable normal tissues of 30 patients. **C** and **D**. Univariate and multivariate cox regression analysis of miR-27b-3p via TCGA database. **E**. The expression of miR-27b-3p in 30 HCC samples based on the expression of circRanGAP1, result revealed lower expression level of miR-27b-3p level in the circRanGAP1^high^ group than that in the circRanGAP1^low^ group. **F–I**. CCK-8, colony formation, transwell migration, and invasion assays were performed in HCCLM3 cells transfected with miR-NC+NC shRNA, NC shRNA+miR-27b-3p inhibitor, miR-27b-3p inhibitor+circRanGAP1 shRNA, circRanGAP1 shRNA+miR-NC, and in Huh7 cells treated with miR-NC+Mock, miR-NC+circRanGAP1, miR-27b-3p mimic+circRanGAP1, miR-27b-3p mimic +Mock. **J** and **K**. Tumor growth in Huh7 cells treated with miR-NC+Mock, miR-NC+circRanGAP1, miR-27b-3p mimic+circRanGAP1, miR-27b-3p mimic +Mock investigated by mice xenograft tumor models. The data are represented as the mean ± SD, n=3. *P < 0.05; **P < 0.01; ***P < 0.001; NS, not significant

According to previous reports, miR-27b-3p functions as a suppressor of tumors, they suppress the progression of breast cancer, gastric cancer, and colorectal cancer [26–28]. Therefore, quantitative real-time PCR was done to evaluate the level of miR-27b-3p in HCC, and the results revealed a dramatically lower concentration of miR-27b-3p in HCC cells than in normal L02 liver cells (Fig. 4A) and matched normal tissues (Fig 4B). This result is identical to previous analytical results from TCGA data. As Additional file 1: S4A, B show, miR-27b-3p levels were significantly low-expressed in HCC tissues as compared to normal matched tissues (Additional file 1: Fig S4A). HCC patients that had less miR-27b-3p expression levels had a poorer prognosis than those with enhanced expression of miR-27b-3p. Furthermore, as presented in Additional file 1: Fig S4C, the cutoff value, sensitivity, specificity, and AUC of miR-27b-3p were 11.507, 0.800, 0.750, and 0.824, respectively, in the TCGA cohort, clarifying that miR-27b-3p is a promising diagnostic biomarker for HCC. Moreover, univariate and multivariate Cox regression analyses show that miR-27b-3p, pathological and TNM stage are independent prognostic factors for poor HCC prognosis (Fig 4C, D). We then investigated its biological role in HCC. The miR-27b-3p inhibitor and mimic decreased and increased its expression in Huh7 and HCCLM3 cells, respectively (Additional file 1: Fig S4D). Its upregulation suppresses the development, invasion, and migration of Huh7 cells, whereas its downregulation in HCCLM3 cells produces the opposite results (Additional file 1: Fig S4E, F), suggesting the function of miR-27b-3p as a tumor suppressor in HCC.

Next, the miR-27b-3p effect on circRanGAP1-mediated oncogenic processes was investigated in in-vitro and in-vivo settings. We further measured miR-27b-3p and circRanGAP1 expression using quantitative real-time PCR in 30 HCC samples. The result revealed that the miR-27b-3p expression level in the circRanGAP1^high^ group was less than that in the circRanGAP1^low^ group (Fig. 4E). Furthermore, circRanGAP1 knockdown-mediated impairment of development, invasion, and migration in HCC cells was partially reversed after miR-27b-3p inhibition (Fig. 4F, H). Whereas, miR-27b-3p upregulation repressed circRanGAP1-induced Huh7 cells proliferation, migration, and invasion (Fig. 4G, I). We established orthotopically implanted intrahepatic HCC models in vivo and found that upregulation of miR-27b-3p reduced circRanGAP1 overexpression-induced increase in both tumor size and the rate of pulmonary metastasis (Fig. 4K, L). Together, these results indicate that circRanGAP1 advances the growth and metastasis of HCC via miR-27b-3p sponging.

### Prediction of the downstream immune-related target gene of miR-27b-3p in HCC

**Fig. 5:**
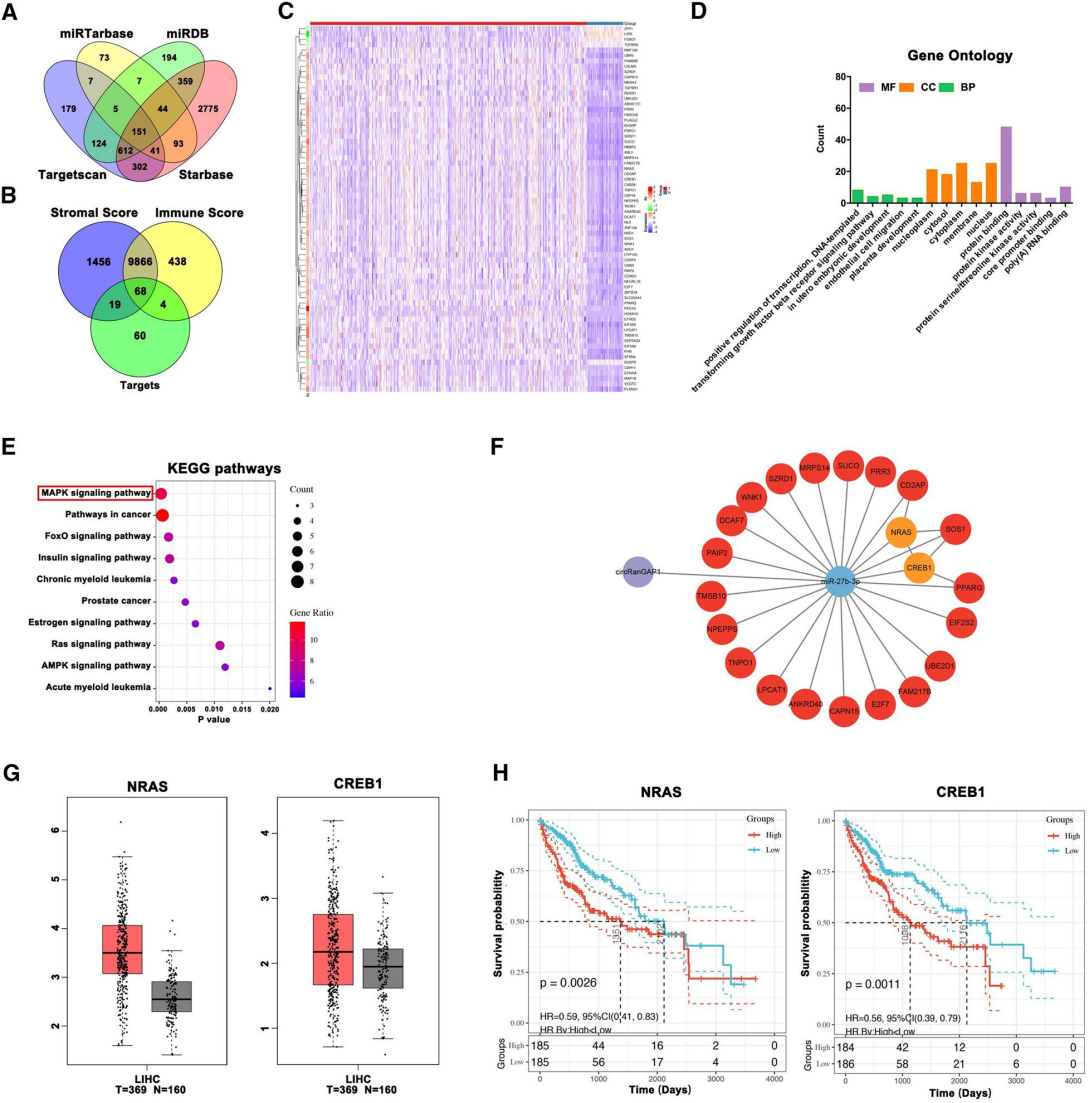
Prediction of the downstream immune-related target gene of miR-27b-3p in HCC. **A**. Venn diagram exhibiting miR-27b-3p target mRNAs overlap, estimated by Targetscan, Starbase, miRDB, and miRTarbase databases. **B**. Overlapped 68 immune-related target genes of miR-27b-3p in 151 predicted targets of miR-27b-3p with the DEGs in the immune and stromal high score group based on the ESTIMATE algorithm. **C**. The heatmap of 68 immune-related miR-27b-3p target genes. **D** and **E**. The top 10 enriched Gene Ontology (GO) and Kyoto Encyclopedia of Genes (KEGG) pathways of 68 immune-related DEGs. **F**. A circRNA-miRNA-mRNA network of 22 genes associated with the poor prognosis of HCC patients via Cytoscape software. Red: upregulation, Orange: upregulation, Green: downregulation. **G**. The NRAS and CREB1 expression in tissues effected with HCC compared with matched adjacent normal tissues from the GEPIA database. **H***.* The survival analyses of NRAS and CREB1. *P < 0.05; **P < 0.01; ***P < 0.001; *NS* not significant

The miRNAs mainly exert their effects on cancer cells by directly targeting mRNAs; therefore, here the downstream miR-27b-3p target genes were also estimated using the Targetscan, Starbase, miRDB, and miRTarbase databases. A total of 151 target genes overlapped among the four databases (Fig. 5A). Subsequently, 68 genes were obtained by intersecting 151 predicted targets of miR-27b-3p with the DEGs in the immune and stromal high-score groups based on the ESTIMATE algorithm (Fig. 5B). The differential expression levels of the 68 genes were depicted in Fig. 5C. Next, 68 immune-related genes were typed into the David database for gene ontology (GO) and Kyoto Encyclopedia of Genes (KEGG) pathway enrichment analysis. The top 10 enriched GO terms and KEGG pathways were shown in Fig. 5D, E, respectively. According to KEGG analysis, the MAPK signaling pathway might be the most potential miR-27b-3p downstream pathway. Moreover, survival analyses of the 68 genes suggested that 22 genes were associated with poor outcomes in HCC (Additional file 1: Fig S5A). To build a network of protein-protein interaction (PPI), 22 genes were uploaded to the STRING database. The Cytoscape software was used to establish the complete circRNA-miRNA-mRNA network. Three algorithms (degree, closeness, and betweenness) were performed to analyze the relationships between genes using the CytoHubba plugin of Cytoscape software. The results showed that NRAS and CREB1 are key genes that play a major role in the entire network, except for miRNAs (red: upregulation, orange: upregulation, green: downregulation) (Fig. 5F and Additional file 1: Fig S5B). NRAS and CREB1 were markedly expressed in tissues of HCC as compared to normal ones according to the GEPIA database (Fig. 5G). In addition, survival analysis showed that NRAS and CREB1 were significantly associated with a poor HCC prognosis (Fig. 5H). Altogether, we may conclude that NRAS and CREB1 act as miR-27b-3p downstream immune targets in HCC.

### circRanGAP1 promotes proliferation, migration, and invasion of HCC cells by regulating miR-27b-3p/NRAS/ERK axis

**Fig. 6:**
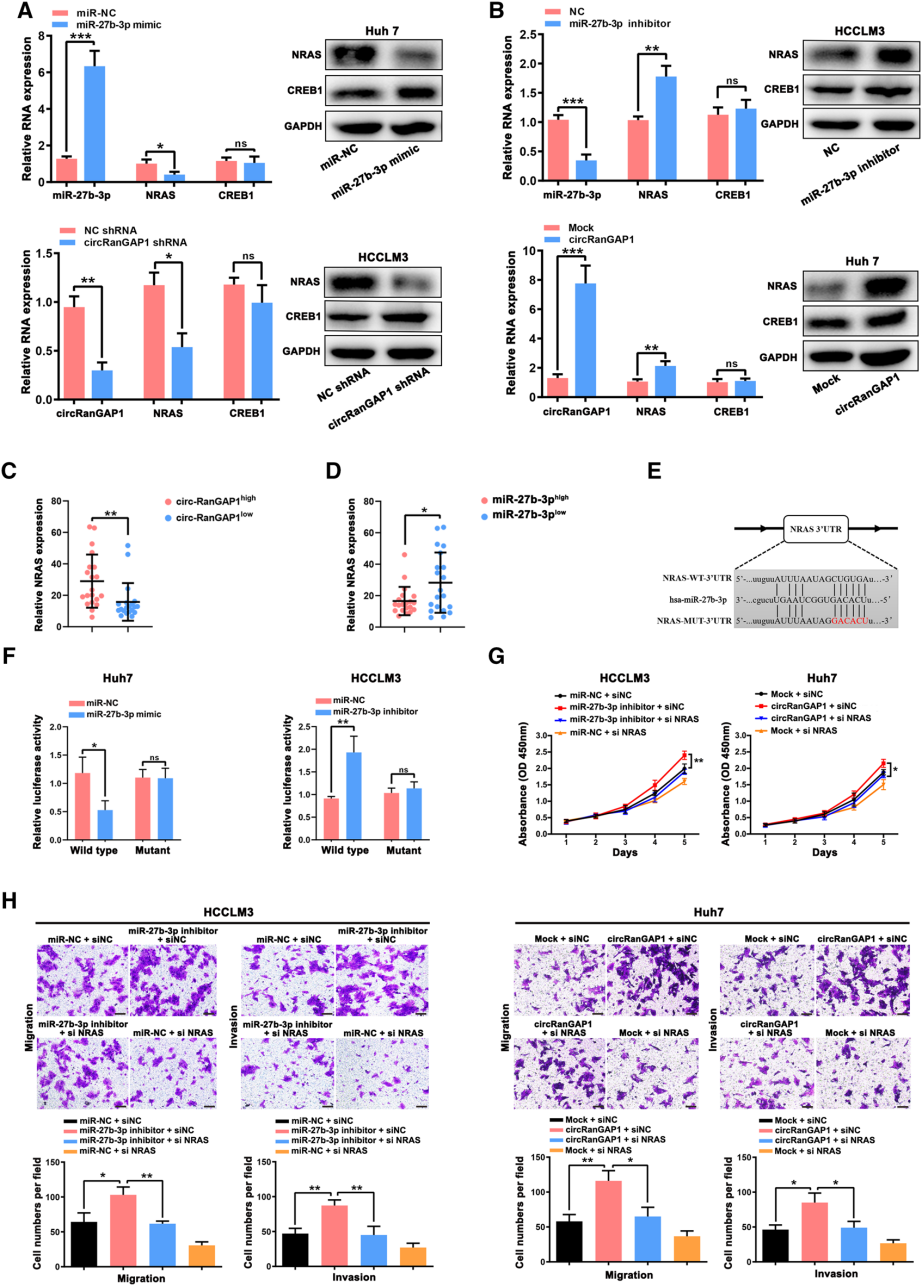
circRanGAP1 promotes HCC cell progression through regulating miR-27b-3p/NRAS axis. **A **and **B**. NRAS and CREB1 mRNA and protein levels in Huh7 cells after miR-27b-3p mimic or circRanGAP1 transfection and in HCCLM3 cells after transfection with miR-27b-3p inhibitor or circRanGAP1 shRNA. **C** and **D**. The NRAS expression in 30 HCC specimens based on circRanGAP1 and miR-27b-3p expression showed that NRAS levels in the circRanGAP1^high^ group were greater than observed in circRanGAP1^low^ group, whereas, NRAS levels in miR-27b-3p^high^ group were less than the miR-27b-3p^low^ group. **E**. The binding sites of miR-27b-3p with the WT or Mut NRAS 3-UTR were predicted using StarBase v3.0. **F**. Luciferase activity of WT or Mut NRAS 3-UTR after transfection with miR-27b-3p mimic or inhibitor in Huh7 and HCCLM3 cells. **G** and **H**. Verification of the effects of miR-27b-3p on NRAS expression in Huh7 and HCCLM3 cells via CCK-8, Transwell migration, and invasion assays. Data are presented as the mean ± SD, n=3. *P < 0.05; **P < 0.01; ***P < 0.001; *NS* not significant

Several studies reported that miRNAs/circRNAs plays important role in the physiological processes of tumor cells by modulating downstream target gene expressions [11, 29, 30]. To verify whether NRAS or CERB1 is one of these genes, quantitative real-time PCR and western blotting were done to evaluate NRAS and CERB1 expression. As presented in Fig. 6A, B, the results revealed a marked reduction in NRAS mRNA and protein levels in miR-27b-3p mimic transfected Huh7 cells or in circRanGAP1-knockdown HCCLM3 cells, while remarkably enhanced in miR-27b-3p inhibitor transfected HCCLM3 cells or in circRanGAP1 over-expressed Huh7 cells, while the CREB1 mRNA and protein levels remained unchanged. These results indicate that NRAS, but not CERB1, could be an important gene target of circRanGAP1/miR-27b-3p. Quantitative real-time PCR analysis of 30 HCC tissues showed that the circRanGAP1^high^ group’s NRAS levels were more than that of the circRanGAP1^low^ group (Fig. 6C), and NRAS levels in the miR-27b-3p^high^ group were decreased as compared with a miR-27b-3p^low^ group (Fig. 6D). In HCC cells to verify the presence of the miR-27b-3p binding site in the 3’ -UTR region of the NRAS mRNA sequence, a luciferase reporter gene assay was performed. Co-transfection in Huh7 and HCCLM3 cells was performed by luciferase reporter vectors consisting of Mut or WT 3’-UTR sequence of NRAS along with miR-27b-3p inhibitor/mimic. The results revealed that WT NRAS 3’-UTR luciferase activity was reduced by a miR-27b-3p mimic in Huh7 cells and was enhanced by miR-27b-3p inhibitor in HCCLM3 cells. In contrast, the luciferase activity of the Mut NRAS 3’-UTR remained unaffected by the miR-27b-3p mimic or inhibitor in Huh7 and HCCLM3 cells (Fig. 6E, F).

To further verify whether circRanGAP1 can promote HCC progression by protecting NRAS from miR-27b-3p-induced downregulation, we used small interfering RNA (siRNA) against NRAS (Additional file 1: Fig S6A) to conduct functional rescue experiments. The results revealed that circRanGAP1 overexpression and downregulation of miR-27b-3p induced development, invasion, and migration are blocked by the knockdown of NRAS (Fig. 6G, H). Moreover, we found that NRAS overexpression could reverse the anti-proliferation, anti-migration and anti-invasion effects induced by cirRanGAP1 knockdown in HCCLM3 cells (Additional file 1: Fig S6B–D). Collectively, these results uncovered that circRanGAP1 induces HCC cell proliferation, migration, and invasion via NRAS.

**Fig. 7:**
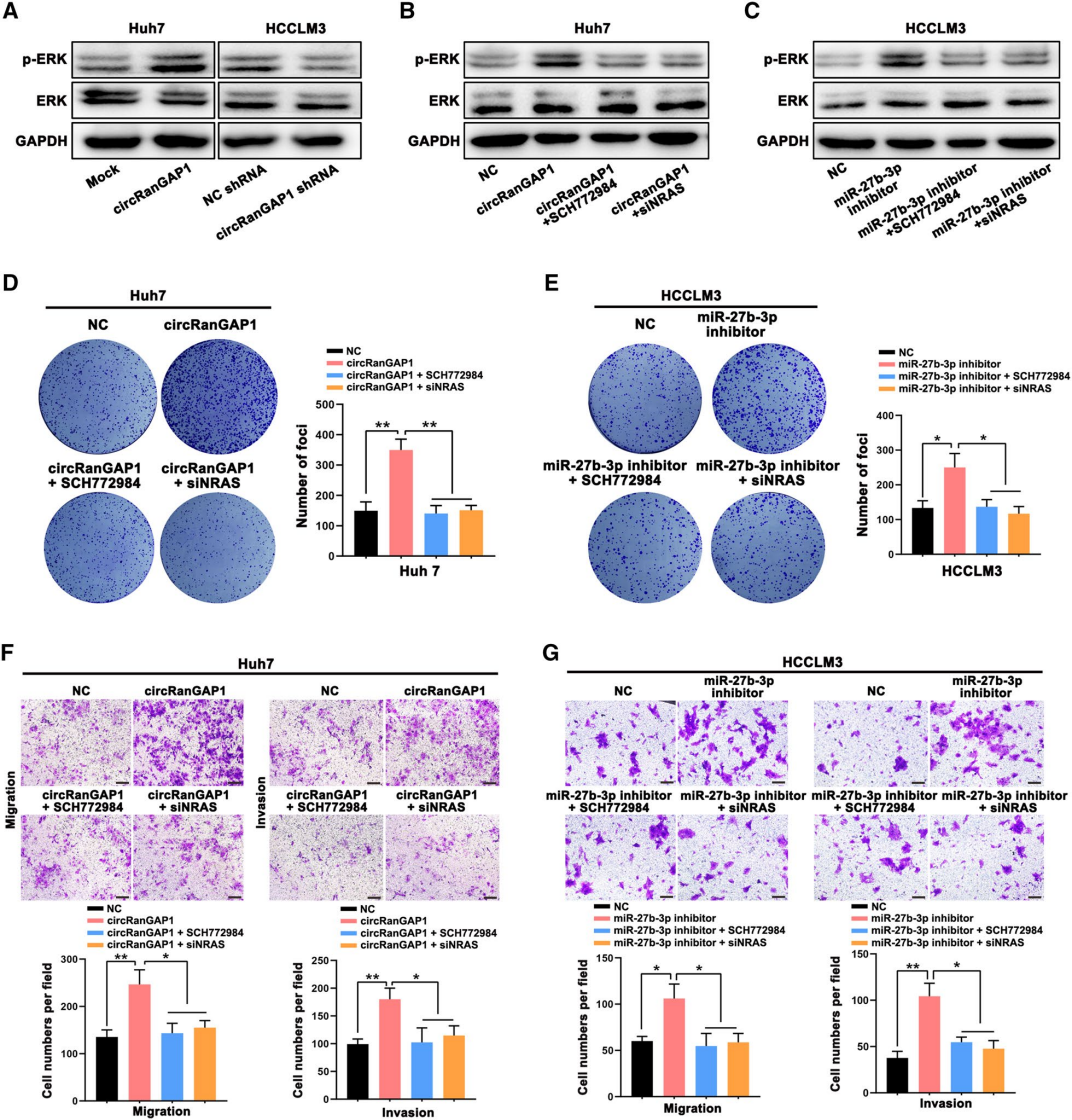
circRanGAP1 promotes HCC cell progression through regulating miR-27b-3p/NRAS/ERK axis. **A**. Western blot assay was carried out for detecting p-ERK, ERK, and GAPDH levels in circRanGAP1-overexpressed Huh 7 cells and circRanGAP1-downregulated HCCLM3 cells and control group cells. As internal control GAPDH was used. **B** and** C**. Western blot assay, performed for evaluating p-ERK, ERK, and GAPDH levels in Huh7 and HCCLM3 cells with different treatments. **D **and **F**. Colony formation, transwell migration, and invasion assays were performed in Huh7 cells to verify the function of ERK inhibitor (SCH772984) and interfering with NRAS expression on the progression of circRanGAP1. **E** and **G**. Colony formation, transwell migration, and invasion assays were performed in HCCLM3 cells to verify the function of ERK inhibitor (SCH772984) and interfering with NRAS expression on the progression of miR-27b-3p inhibitor. The data are represented as the mean ± SD, n=3. *P < 0.05; **P < 0.01; ***P < 0.001; NS, not significant

Our analyses suggested that the MAPK signaling pathway predominantly enhances the miR-27b-3p downstream pathway (Fig. 5E). Therefore, we assessed the activation of the MAPK signaling pathway (ERK phosphorylation) by western blotting. We found that overexpression of circRanGAP1 potentiated the ERK phosphorylation in Huh7 cells, while knockdown of circRanGAP1 suppressed the ERK phosphorylation in HCCLM3 cells (Fig. 7A). The ERK phosphorylation induced by circRanGAP1 may be reversed by treatment with an ERK inhibitor (SCH772984) or by interference with NRAS expression (Fig. 7B). Likewise, rescue experiments verified that the ERK inhibitor or interference with NRAS expression also inhibits the phosphorylation of ERK promoted by the inhibitor of miR-27b-3p (Fig. 7C), suggesting that circRanGAP1 regulates ERK phosphorylation through miR-27b-3p/NRAS axis. Moreover, the data from colony formation and transwell assays revealed that circRanGAP1 accelerates HCC cell growth, migration, and invasion by miR-27b-3p/NRAS/ERK axis regulation (Fig. 7D–G).

### CircRanGAP1 is correlated with tumor-associated-macrophage (TAM) infiltration

**Fig. 8:**
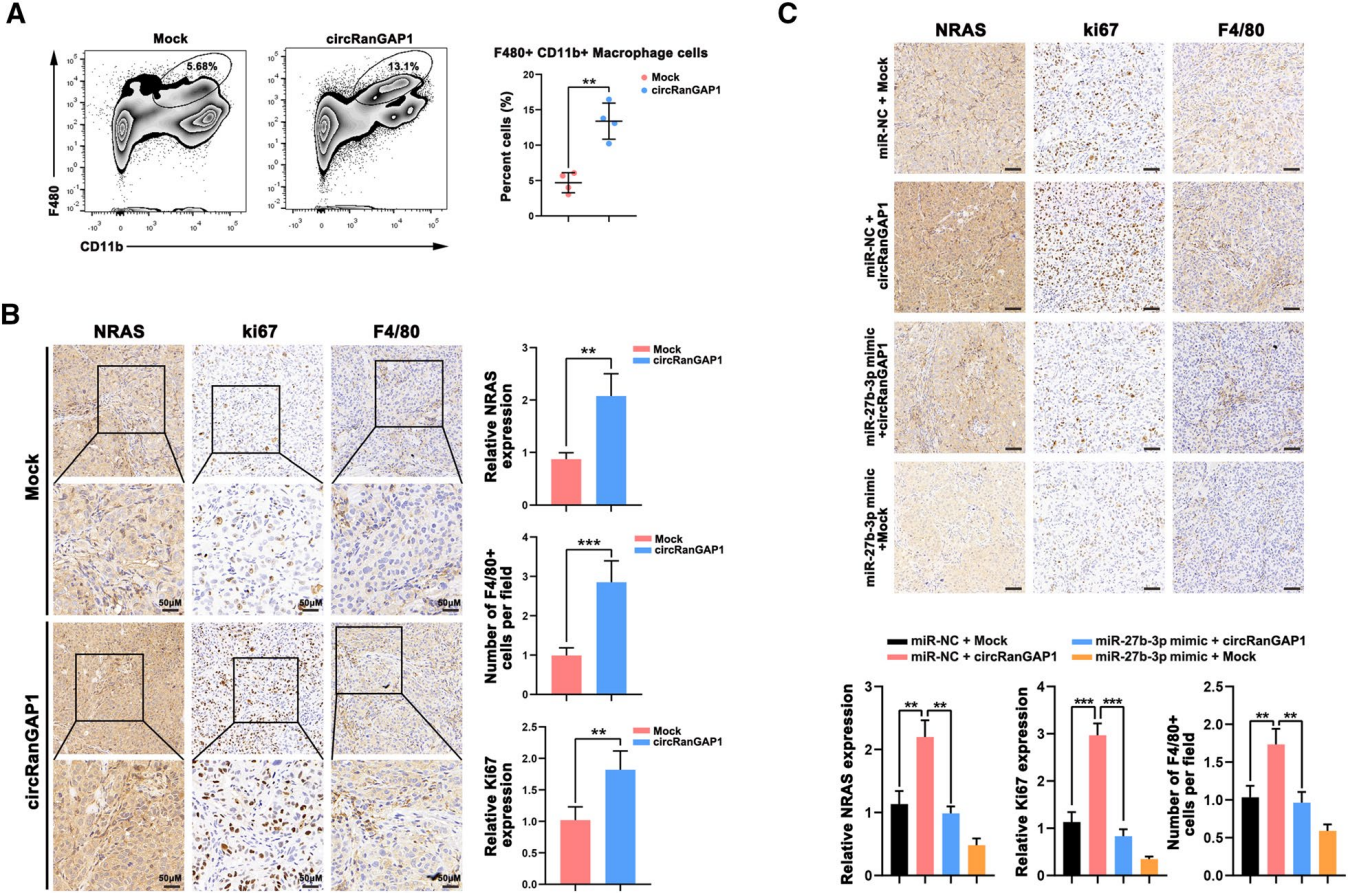
circRanGAP1 is correlated with tumor-associated-macrophage (TAM) infiltration. **A**. Fluorescence-activated cell sorting (FACS) analysis to confirm the infiltration of F4/80+ TAMs in Hep1-6-circRanGAP1 xenografts and Hep1-6-Mock xenografts via a subcutaneous xenograft tumor model. **B**. Confirmed the NRAS expression, ki67 expression, and F4/80+ TAMs infiltration in the Hep1-6-circRanGAP1 group compared with that in the Hep1-6-Mock group through IHC analysis. **C.** IHC analysis was performed for detecting the expression level of NRAS, ki67 expression, and F4/80+ TAMs infiltration after treating with miR-27b-3p mimic. *P < 0.05; **P < 0.01; ***P < 0.001; *NS* not significant

The related immune cells infiltration miR-27b-3p/NRAS level in HCC was assessed using the ssGSEA algorithm in the “GSVA” R package [31, 32]. The results revealed that dendritic cells, T helper cells, macrophages, and Tcm are positively correlated with NRAS, whereas they are negatively correlated with miR-27b-3p (Additional file 1: Fig. S7A–C). For determining the circRanGAP1 effects on immunocyte infiltration, a subcutaneous xenograft tumor model in C57BL/6 mice was established. The infiltration of F4/80+ TAMs in Hep1-6-circRanGAP1 xenografts was found to be greater than that in Hep1-6-Mock xenografts, as evidenced by fluorescence-activated cell sorting (FACS) analysis (Fig. 8A). CircRanGAP1 did not alter the number of infiltrating T cells, NK cells, Treg cells, B cells, and dendritic cells (Additional file 1: Fig. S8A–D). In addition, IHC analysis demonstrated that NRAS expression, ki67 expression, and F4/80+ TAMs infiltration are upregulated in the Hep1-6-circRanGAP1 group in comparison with the Hep1-6-Mock group (Fig. 8B). Besides, the upregulation of miR-27b-3p reduces circRanGAP1-induced NRAS upregulation, F4/80+ TAM infiltration, and ki67 upregulation (Fig. 8C), indicating that circRanGAP1 may promote TAM infiltration probably by sponging miR-27b-3p.

### NRAS+ and CD68+ TAMs have prognostic value for patients with HCC

**Fig. 9:**
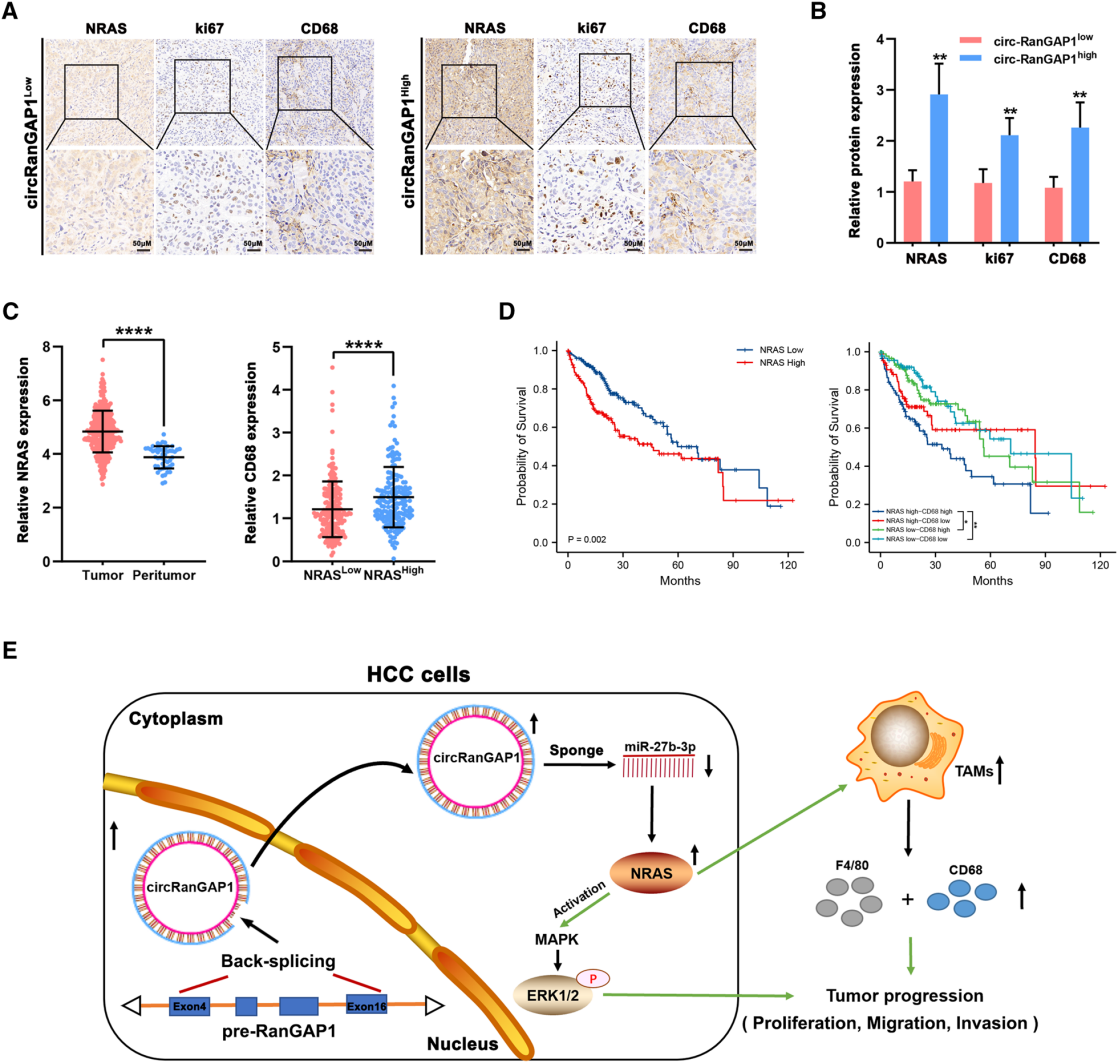
NRAS+ and CD68+ TAMs have prognostic value for patients with HCC. **A** and **B**. IHC analysis showed that patients with high circRanGAP1 expression had high ki67, NRAS, and CD68 protein expression, whereas those with low circRanGAP1 expression had low ki67, NRAS, and CD68 protein expression. **C**. TCGA data reveal the expression of NRAS and the relationship between NRAS and CD68 in HCC tissues. **D**. Kaplan-Meier analysis reveals the prognostic value of NRAS and CD68+ TAMs. **E**. The proposed model illustrates the circRanGAP1–miR-27b-3p/NRAS signaling function in the regulation of HCC progression

To explore the association between circRanGAP1, NRAS, ki67, and CD68+ TAMs in HCC, their expression levels in 15 HCC tissues were measured using quantitative real-time PCR and IHC assays. Representative cases are shown in Fig. 9A. HCC with high circRanGAP1 has high levels of NRAS, ki67 expression, and increased TAM infiltration. Consistent with this, HCC with low circRanGAP1 has low expression of NRAS, ki67, and less TAM infiltration. Correlation analysis of 15 HCC tissues indicated that the level of CD68 + TAMs in the circRanGAP1 ^high^ group was greater compared to the circRanGAP1 ^low^ group (Fig. 9B). Similarly, the level of CD68+ TAMs in the NRAS ^high^ group was more than in NRAS ^low^ group. TCGA data analysis confirmed the enhanced expression of NRAS in HCC tissues rather than in normal control, and that CD68+ TAMs level in the NRAS ^high^ group is markedly increased when compared with that in the NRAS ^low^ group (Fig. 9C). Furthermore, the survival analyses suggested that a higher NRAS expression in HCC patients predicts a worse prognosis. Moreover, we further assessed the combined effects of NRAS and TAMs on the outcome of patients with HCC and found a lower OS rate in NRAS ^high^ CD68 ^high^ patients than in NRAS ^low^ CD68 ^high^ patients (Fig. 9D). Together, these results suggest that CD68+ TAMs and NRAS**+** have a prognostic value in HCC patients.

## Discussion

Increasing evidence elucidated that circRNAs regulate the function of genes and affect human cancer progression [33, 34]. And it was reported that aberrantly expressed circRNAs exist in diverse cancers, such as hepatocellular carcinoma [35], gastric cancer [36], breast cancer [37], lung cancer [38], esophageal cancer [39], etc. Moreover, circRNAs were also found to be involved in various physiological processes. For example, circ_104348 was found to promote the HCC progression by targeting miR-187–3p/RTKN2 axis and activating Wnt/β-catenin pathway [40]. Meanwhile, Xiaojing Li et.al revealed that the loss of NUDT21 prevented circRNA cyclization, resulting in abnormal proliferation of HCC cells [41]. All these results tell that circRNAs may be potential biomarkers in HCC. Unfortunately, few studies specified their roles in the correlation between HCC progression and the altered immune microenvironment. Here, we examined 5 RanGAP1-derived circRNAs, and among them, circ_0063513 is the highest expressed circRNA in tissues with HCC than in those which are physiologically normal. We further investigated the correlation between clinicopathological manifestations and circRanGAP1 in HCC patients. Our data revealed that the expression level of circRanGAP1 was highly linked with enlarged tumors and advanced stages of TNM. Mechanistically, we identified that circ_0063513 can induce HCC development by regulating miR-27b-3p/NRAS/ERK axis in combination with bioinformatics analyses and in vitro and in vivo, and immune infiltration-related analyses revealed that circRanGAP1 can also affect the infiltration level of tumor-associated macrophages. Our study can provide advancement in the domain of non-coding RNA research in HCC.

Recently, several circRNAs were identified as oncogenes in HCC. For instance, has_ circ_0091581 can accelerate the deterioration of HCC [42], and has_circ_0003645 enhances HCC cell proliferation, metastasis, and invasion [43]. Using bioinformatics technology, and combining with relevant databases (e.g., StarBase v2.0, TarBase v.8, and GEPIA) and in vitro and in vivo, we found that elevated circRanGAP1 may serve as an oncogene to accelerate the HCC progression.

CircRNAs, as endogenous RNAs (ceRNAs), are known to act as sponge miRNAs, thus reversing their function [44]. For example, Pan H et. al showed that circUBA1 advances the development and metastasis of gastric tumor cells by sponging miR-375 [45]. Previous researchers have discovered that both hsa_circ0000885 and circPGD can promote cancer progression via sponging miR-16-5p [46, 47]. Consistently, in our study, we found that circRANGAP1 promotes HCC progression by sponging miR-27b-3p. Previous studies revealed that miR-27b-3p is involved in multiple cancers. It can inhibit the translation of many genes into proteins, causing the abnormal function of a large number of genes [26]. Commonly, it generally plays a role in anti-cancer genes in tumors [48–50]. In this investigation, we found that miR-27b-3p inhibits HCC cell proliferation and invasion. Similar to this finding, Zhu et al verified that miR-27b-3p is abnormally upregulated in HCC [51]. Besides, the role of miR-27b-3p is highly comparable not only in HCC but also in other tumors [27, 28, 52]. Furthermore, Wu et al found that miR-27b-3p downregulation causes chemoresistance in colorectal cancer (CRC), revealing the anti-cancer effect of miR-27b-3p in CRC [26]. Moreover, it was reported that it also suppresses the development and invasion of breast cancer cells, as well as increases the sensitivity to chemotherapeutic drugs [27]. These studies provide a rich theoretical basis as well as feasibility for circRanGAP1 and miR-27b-3p interactions to regulate HCC progression.

NRAS is a RAS family member, that works as a switch regulating GDP/GTP, which encodes GTPase (membrane-bound protein) activity and is a key regulatory factor in multiple biological processes that affect cancer survival and progression [53]. And the RAS/RAF/ERK (mitogen-activated protein kinase/ERK) is found to be hyperactivated in cancers and is capable of promoting a malignant phenotype of tumors [54]. More importantly, the relationship between NRAS and MAPK signaling in tumors has been also reported before, such as Christian Adam et al found that the co-inhibition of ERK1/2 and ERK5 MAPK pathways can effectively suppress NRAS-driven melanoma [55], and in addition, Wei Song et al verified that HBx mediated TRERNA1 elevation leads to resistance of sorafenib and HCC cell proliferation through miR-22-3p/NRAS axis [56]. Combining these prior studies provides a wealth of theoretical knowledge for our study and increases the feasibility of the experiments.

To study the oncogenic role of has_circ_0063513 in HCC, we utilized a bioinformatics tool to perform a KEGG pathway enrichment analysis. Bioinformatic analyses have been widely used in various diseases, including gastric cancer [57], kidney renal clear cell carcinoma [58], and lung adenocarcinoma [59]. etc. In our previous study, bioinformatics analysis showed that the ITGB1-DT/ARNTL2 axis induces lung adenocarcinoma progression [60], suggesting the important role of bioinformatics. Moreover, the application of bioinformatic analysis can enhance our understanding of research at the molecular level [61]. And now, the results of KEGG revealed that the MAPK signaling (ERK) pathway might be the circRanGAP1/miR-27b-3p/NRAS axis targeting pathway, and the result was also elucidated through western blotting.

With the development of immune microenvironment research and technology, a strong link between immune infiltration and tumor progression has been found [62, 63]. For example, Anderson et al. [64] found that the functional plasticity of macrophages as immune effector cells leads to their antitumor and pro-tumor functions in different microenvironments. Macrophage diversity has also been reported to enhance tumor progression and metastasis [65]. Therefore, we used the ssGSEA immune algorithm to analyze tumor purity in tumor tissues to screen for immune cells that may affect tumor progression [31, 32]. We found that the circRanGAP1/miR-27b-3p/NRAS axis may be responsible for macrophage infiltration in HCC. To verify whether circRanGAP1 can alter the immune microenvironment, we established a subcutaneous xenograft tumor model to further assess the immune cell infiltration level. Ultimately, our study found that circRanGAP1 could regulate TAMs infiltration in HCC.

## Conclusion

In conclusion, we demonstrated that circRanGAP1 (hsa_circ_0063513) is elevated in HCC and that a high level of circRanGAP1 is related to enlarged tumor dimensions and terminal TNM stage in HCC patients. Moreover, we elucidated the potential mechanism by which circRanGAP1 accelerates the HCC progression by modulating the miR-27b-3p/NRAS/ERK axis and influencing the infiltration levels of tumor-associated macrophages. All in all, our study not only elucidated a potential mechanism highlighting how circRNAs affect HCC progression and immune infiltration but also promulgated that circRANGAP1/ NRAS axis may be an important therapeutic target for HCC patients.

## Supplementary Information

**Additional file 1: Figure S1.** Characterization of circRanGAP1 in HCC. **Figure S2.** circRanGAP1 expression in HCC cells. **Figure S3.** CircRanGAP1 acted as a sponge of miR-27b-3p in HCC cells. **Figure S4.** miR-27b-3p plays tumor-suppressive roles in HCC. **Figure S5.** **Figure S6.** The effect of NRAS in cirRanGAP1 knockdown cells. **Figure S7.** Immune infiltration analysis of miR-27b-3p and NRAS. **Figure S8.** The effects of circRanGAP1 on immunocytes infiltration. **Table S1.** Sequences of Primers used for qRT-PCR. **Table S2.** List of Primary Antibodies Used in the Study. **Table S3.** Target sequences of hsa_circ_0063513 shRNAs. **Table S4.** circ_0063513 and miR-27b-3p FISH probe sequences. **Table S5.** The potential targets of RanGAP1.

## Data Availability

The datasets used and/or analyzed during the current study are available from the corresponding author on reasonable request.
