# Supplementary material for: circRanGAP1/miR-27b-3p/NRAS Axis may promote the progression of hepatocellular Carcinoma
Source: Exp Hematol Oncol. 2022 Nov 8;11:92. doi: 10.1186/s40164-022-00342-6 (PMC9644583; doi:10.1186/s40164-022-00342-6)
Supplement: Supplementary file 1 — Additional file 1: Figure S1. Characterization of circRanGAP1 in HCC. Figure S2. circRanGAP1 expression in HCC cells. Figure S3. CircRanGAP1 acted as a sponge of miR-27b-3p in HCC cells. Figure S4. miR-27b-3p plays tumor-suppressive roles in HCC. Figure S5. Figure S6. The effect of NRAS in cirRanGAP1 knockdown cells. Figure S7. Immune infiltration analysis of miR-27b-3p and NRAS. Figure S8. The effects of circRanGAP1 on immunocytes infiltration. Table S1. Sequences of Primers used for qRT-PCR. Table S2. List of Primary Antibodies Used in the Study. Table S3. Target sequences of hsa_circ_0063513 shRNAs. Table S4. circ_0063513 and miR-27b-3p FISH probe sequences. Table S5. The potential targets of RanGAP1. [file 40164_2022_342_MOESM1_ESM.pdf]

Supplementary Figure legend

Supplementary Figure 1 Characterization of circRanGAP1 in HCC

A. Scheme illustrating the production of hsa_circ_0063513 (termed circ-RanGAP1) via exons 4–16 circularization of the RanGAP1 gene. **B.** Random hexamer or oligo (dT)18 primers were used in the reverse transcription experiments. The relative RNA levels were analysed by RT-qPCR and normalized to the value using random hexamer primers in HCC-derived Huh7 and HCCLM3 cells. **C.** qRT-PCR analysis of the expression levels of circRanGAP1 and RanGAP1 after treatment with RNase R in Huh7 and HCCLM3 cells. **D.** The relative RNA levels of circRanGAP1 and RanGAP1 were analysed by RT-qPCR after treatment with Actinomycin D at the indicated time points in Huh7 and HCCLM3 cells. **E.** circRanGAP1 and RanGAP1 are abundant in the cytoplasm of Huh7 and HCCLM3 cells. GAPDH and U2 were applied as positive controls in the cytoplasm and nucleus, respectively. **F.** RNA FISH for circRanGAP1 in Huh7 and HCCLM3 cells. Nuclei were stained with DAPI. Scale bar, 10μm. The data are represented as the mean ± SD, n=3. *P < 0.05; **P < 0.01; ***P < 0.001; NS, not significant.

Supplementary Figure 2 circRanGAP1 expression in HCC cells

A. circRanGAP1 expression in six HCC cell lines and normal liver cell L02 was examined using qRT-PCR analysis. **B and C.** The efficacy of circRanGAP1 shRNA 1-3 in HCCLM3 and HepG2 cells was analyzed by qRT-PCR. **D and E.** The efficacy of circRanGAP1 overexpression in MHCC97H and Huh7 cells and interference in HCCLM3 and HepG2 cells was analyzed by qRT-PCR. **F.** RanGAP1 expression in circRanGAP1 knockdown HCC cells. The data are represented as the mean ± SD, n=3. *P < 0.05; **P < 0.01; ***P < 0.001; NS, not significant.

Supplementary Figure 3 CircRanGAP1 acted as a sponge of miR-27b-3p in HCC cells

A. Online circular RNA interactome (https://circinteractome.nia.nih.gov/index.html) revealed Ago2 occupancy in the region of circRanGAP1. **B.** The expression of miR-27b-3p in Huh7 and HCCLM3 cells after transfection with circRanGAP1 or circRanGAP1 shRNA. **C.** The expression of circRanGAP1 in Huh7 and HCCLM3 cells

after transfection with miR-27b-3p mimic or miR-27b-3p inhibitor. The data are represented as the mean ± SD, n=3. *P < 0.05; **P < 0.01; ***P < 0.001; NS, not significant.

Supplementary Figure 4 miR-27b-3p plays tumor-suppressive roles in HCC

A. Analysis of the expression of miR-27b-3p in HCC tissues and matched adjacent nontumor tissues by TCGA database. **B.** The TCGA database was used to analyze the overall survival of miR-27b-3p in HCC patients. **C.** ROC curve analysis of the cutoff value, sensitivity, specificity, and AUC of miR-27b-3p in HCC tissues from TCGA data. **D.** The efficacy of miR-27b-3p after transfection with miR-27b-3p mimic or miR-27b-3p inhibitor analyzed by qRT-PCR. **E and F.** CCK-8, transwell migration, and invasion assays were performed in HCC and control cells after transfection with miR-27b-3p mimic or miR-27b-3p inhibitor. The data are represented as the mean ± SD, n=3. *P < 0.05; **P < 0.01; ***P < 0.001; NS, not significant.

Supplementary Figure 5

A. The survival analyses of 22 immune-related DEGs. **B.** Three algorithms (degree, closeness, betweenness) were performed to study and analyze the relationships between genes by using the CytoHubba plugin of Cytoscape software.

Supplementary Figure 6 The effect of NRAS in cirRanGAP1 knockdown cells

A. Huh7 and HCCLM3 cells were transfected respectively with three different small interfering RNA (siRNA) targeting NRAS mRNA to identify the most efficient sequence of siRNA by qRT-PCR and western blot. **B and C and D.** NRAS overexpression reversed the anti-proliferation, anti-migration and anti-invasion effects induced by cirRanGAP1 knockdown in HCCLM3 cells via CCK-8, Transwell migration, and invasion assays. The data are represented as the mean ± SD, n=3. *P < 0.05; **P < 0.01; ***P < 0.001; NS, not significant.

Supplementary Figure 7 Immune infiltration analysis of miR-27b-3p and NRAS

A. The immune infiltration analyses of miR-27b-3p and NRAS were performed using the ssGSEA algorithm in the "GSVA" R package. **B.** Intersecting the correlated immune cells of miR-27b-3p and NRAS. **C.** The correlation analyses between dendritic cells, T helper cells, macrophages, Tcm and miR-27b-3p, NRAS using the Pearson correlation

analysis. *P < 0.05; **P < 0.01; ***P < 0.001; NS, not significant.

Supplementary Figure 8 The effects of circRanGAP1 on immunocytes infiltration

A-D. Fluorescence-activated cell sorting (FACS) analyses were performed to confirm the infiltration of T cells, Treg cells, NK cells, B cells, and dendritic cells in Hep1-6-circRanGAP1 xenografts and Hep1-6-Mock xenografts via a subcutaneous xenograft tumor model. *P < 0.05; **P < 0.01; ***P < 0.001; NS, not significant.

Supplementary Table 1. Sequences of Primers used for qRT-PCR.

Primer name	Sequence 5'-3'
hsa_circ_0063507	Forward: TCGTGTGGCAGCTTCAGCC
	Reverse: GGGATGGGAGAGGCTTTGAGTC
hsa_circ_0063513	Forward: GGTAGGGTCTGGACTGGGCA
	Reverse: ACTCCAGTGGCAGCGTGAAG
hsa_circ_0063518	Forward: TGGACGCTGCTCTTAAAGGAC
	Reverse: CAGGTGACGGGGAAAGTGAA
hsa_circ_0063531	Forward: CATGCCACAGAATGGGATCA
	Reverse: AGAGCTGCAGCCAGGATCTC
hsa_circ_0063534	Forward: GTGGAAGCAGCCAGGGTCAT
	Reverse: CCCTGGAGATCTGCAGACAGG
hsa_circ_0003723	Forward: GGAGCTGGACTTAAGCGACAA
	Reverse: CCCTGGAGATCTGCAGACTTG
CircANRIL	Forward: AGAGAGAATTTTGACAGTGTC
	Reverse: CCAGCACACCTAACAGTGATG
RanGAP1	Forward: GATGGCGCTGAACCACATGG
	Reverse: AAGGAGCAGGATTCCAGGGC
NRAS	Forward: GAAACCTCAGCCAAGACCAGACAG
	Reverse: TCCCATACAACCCTGAGTCCCATC
GAPDH	Forward: GGTATGACAACGAATTTGGC
	Reverse: GAGCACAGGGTACTTTATTG

Supplementary Table 2. List of Primary Antibodies Used in the Study.

Antibody	Applications	Company
NRAS	WB, IHC	Santa Cruz Biotechnology (sc-31)
CREB1	WB	Santa Cruz Biotechnology (sc-271)
GAPDH	WB	Abcam (ab8245)
p-ERK	WB, IHC	Abcam (ab17942)
ERK	WB, IHC	CST (#8544)
AGO2	RIP	Abcam (ab32381)
IgG	RIP	Abcam (ab172730)
CD68	IHC	Abcam (ab213363)
Ki67	IHC	Abcam (ab15580)
F480	IHC	CST (#70076)
CD45	Fc	Biolegend (103134)
CD3	Fc	eBioscience (25-0031-82)
CD4	Fc	eBioscience (11-0042-82)
CD8	Fc	eBioscience (45-0081-82)
CD25	Fc	eBioscience (17-0251-82)
FoxP3	Fc	eBioscience (12-5773-82)
NK1.1	Fc	eBioscience (17-5941-82)
B220	Fc	eBioscience (25-0452-821
MHC-II	Fc	Biolegend (107606)
CD11c	Fc	eBioscience (12-0114-82)
F480	Fc	Biolegend (123137)
CD11b	Fc	eBioscience (45-0112-82)

Abbreviations: CST: Cell Signaling Technology; WB, western blot; IHC, immunohistochemistry; Fc, flow cytometric analysis; RIP, RNA immunoprecipitation.

Supplementary Table 3. Target sequences of hsa_circ_0063513 shRNAs.

shRNA	Target sequence
circ_0063513	
shRNA-1	TCGTGTGGCAGCTTCACGCTG
shRNA-2	CAGCTTCACGCTGCCACTGGA
shRNA-2	GCAGCTTCACGCTGCCACTGG

Supplementary Table 4. circ_0063513 and miR-27b-3p FISH probe sequences.

Name	Sequence
circ_0063513 FISH probe	ACTCCAGTGGCAGCGTGAAGCTGCCACACG
miR-27b-3p FISH probe	gCAGAACTTAGCCACTGTGAa

Supplementary Table 5: The potential targets of RanGAP1

Database	miRNA
starBase	hsa-miR-22-3p<br>hsa-miR-3611<br>hsa-miR-1321<br>hsa-miR-4756-5p<br>hsa-miR-4739<br>hsa-miR-485-3p<br>hsa-miR-539-3p<br>hsa-miR-455-3p<br>hsa-miR-324-3p<br>hsa-miR-1913<br>hsa-miR-665<br>hsa-miR-2278<br>hsa-miR-503-5p<br>hsa-miR-5047<br>hsa-miR-1301-3p<br>hsa-miR-2355-5p<br>hsa-miR-505-3p<br>hsa-miR-6512-3p<br>hsa-miR-6720-5p<br>hsa-miR-4726-5p<br>hsa-miR-4640-5p<br>hsa-miR-3690<br>hsa-miR-346<br>hsa-miR-214-5p<br>hsa-miR-296-3p<br>hsa-miR-216a-3p<br>hsa-miR-128-3p<br>hsa-miR-3681-3p<br>hsa-miR-1193<br>hsa-miR-542-3p<br>hsa-miR-1286<br>hsa-miR-3186-3p<br>hsa-miR-149-5p<br>hsa-miR-605-3p<br>hsa-miR-491-5p<br>hsa-miR-3127-5p<br>hsa-miR-3918<br>hsa-miR-339-3p<br>hsa-miR-129-1-3p<br>hsa-miR-129-2-3p<br>hsa-miR-331-3p<br>hsa-miR-650

	hsa-miR-3612<br>hsa-miR-510-5p<br>hsa-miR-4436a<br>hsa-miR-5000-3p<br>hsa-miR-1197<br>hsa-miR-642b-3p<br>hsa-miR-642a-3p<br>hsa-miR-3173-5p<br>hsa-miR-6799-3p<br>hsa-miR-2114-5p<br>hsa-miR-627-5p<br>hsa-miR-195-5p<br>hsa-miR-497-5p<br>hsa-miR-15a-5p<br>hsa-miR-16-5p<br>hsa-miR-15b-5p<br>hsa-miR-424-5p<br>hsa-miR-6838-5p<br>hsa-miR-214-3p<br>hsa-miR-3619-5p<br>hsa-miR-761<br>hsa-miR-6893-3p<br>hsa-miR-370-3p<br>hsa-miR-182-5p<br>hsa-miR-151b<br>hsa-miR-151a-5p<br>hsa-miR-6807-3p<br>hsa-miR-217<br>hsa-miR-27a-3p<br>hsa-miR-27b-3p<br>hsa-miR-425-5p<br>hsa-miR-378g<br>hsa-miR-942-5p<br>hsa-miR-516b-5p<br>hsa-miR-6763-5p<br>hsa-miR-3150a-3p<br>hsa-miR-199a-5p<br>hsa-miR-199b-5p<br>hsa-miR-670-3p<br>hsa-miR-212-5p<br>hsa-miR-3187-3p<br>hsa-miR-653-5p<br>hsa-miR-1306-5p<br>hsa-miR-506-5p

	hsa-miR-103a-3p<br>hsa-miR-107<br>hsa-miR-324-5p<br>hsa-miR-642a-5p<br>hsa-miR-432-5p<br>hsa-miR-34b-5p<br>hsa-miR-2682-5p<br>hsa-miR-449c-5p<br>hsa-miR-588<br>hsa-miR-4701-5p<br>hsa-miR-1249-3p<br>hsa-miR-449a<br>hsa-miR-449b-5p<br>hsa-miR-34a-5p<br>hsa-miR-34c-5p<br>hsa-miR-876-5p<br>hsa-miR-3167<br>hsa-miR-4664-3p<br>hsa-miR-365a-3p<br>hsa-miR-365b-3p<br>hsa-miR-526b-5p<br>hsa-miR-138-5p<br>hsa-miR-668-3p<br>hsa-miR-1245b-5p<br>hsa-miR-3142<br>hsa-miR-6504-5p<br>hsa-miR-3064-5p<br>hsa-miR-6823-3p<br>hsa-miR-2114-3p<br>hsa-miR-216b-5p<br>hsa-miR-421<br>hsa-miR-212-3p<br>hsa-miR-132-3p<br>hsa-miR-500a-3p<br>hsa-miR-520a-5p<br>hsa-miR-525-5p<br>hsa-miR-374a-5p<br>hsa-miR-374b-5p<br>hsa-miR-1323<br>hsa-miR-548o-3p<br>hsa-miR-299-3p<br>hsa-miR-577<br>hsa-miR-5590-3p<br>hsa-miR-142-5p

	hsa-miR-193a-5p<br>hsa-miR-382-5p<br>hsa-miR-4761-5p<br>hsa-miR-3194-5p<br>hsa-miR-6783-3p<br>hsa-miR-1343-3p<br>hsa-miR-769-5p<br>hsa-miR-376a-3p<br>hsa-miR-376b-3p<br>hsa-miR-130a-5p<br>hsa-miR-23a-3p<br>hsa-miR-23b-3p<br>hsa-miR-23c<br>hsa-miR-382-3p<br>hsa-miR-708-5p<br>hsa-miR-28-5p<br>hsa-miR-3139<br>hsa-miR-1252-5p<br>hsa-miR-296-5p<br>hsa-miR-362-3p<br>hsa-miR-329-3p<br>hsa-miR-582-5p<br>hsa-miR-345-3p<br>hsa-miR-532-3p<br>hsa-miR-326<br>hsa-miR-330-5p<br>hsa-miR-944<br>hsa-miR-126-5p<br>hsa-miR-1287-5p<br>hsa-miR-545-5p<br>hsa-miR-144-5p<br>hsa-miR-374b-3p<br>hsa-miR-4661-5p<br>hsa-miR-139-5p<br>hsa-miR-10a-5p<br>hsa-miR-10b-5p<br>hsa-miR-152-3p<br>hsa-miR-148b-3p<br>hsa-miR-148a-3p<br>hsa-miR-4295<br>hsa-miR-454-3p<br>hsa-miR-3666<br>hsa-miR-130b-3p<br>hsa-miR-130a-3p

	hsa-miR-301a-3p<br>hsa-miR-301b-3p<br>hsa-miR-342-3p<br>hsa-miR-494-3p<br>hsa-miR-3940-3p<br>hsa-miR-760<br>hsa-miR-3194-3p<br>hsa-miR-1266-3p<br>hsa-miR-30a-5p<br>hsa-miR-30d-5p<br>hsa-miR-30b-5p<br>hsa-miR-30e-5p<br>hsa-miR-30c-5p<br>hsa-miR-625-5p<br>hsa-miR-3196<br>hsa-miR-3180<br>hsa-miR-6816-5p<br>hsa-miR-3180-3p<br>hsa-miR-1908-5p<br>hsa-miR-6787-5p<br>hsa-miR-663a<br>hsa-miR-184<br>hsa-miR-127-3p<br>hsa-miR-5586-5p<br>hsa-miR-516a-5p<br>hsa-miR-22-3p<br>hsa-miR-3611<br>hsa-miR-1321<br>hsa-miR-4756-5p<br>hsa-miR-4739<br>hsa-miR-485-3p<br>hsa-miR-539-3p<br>hsa-miR-455-3p<br>hsa-miR-324-3p<br>hsa-miR-1913<br>hsa-miR-665<br>hsa-miR-2278<br>hsa-miR-503-5p<br>hsa-miR-5047<br>hsa-miR-1301-3p<br>hsa-miR-2355-5p<br>hsa-miR-505-3p<br>hsa-miR-6512-3p<br>hsa-miR-6720-5p

	hsa-miR-4726-5p<br>hsa-miR-4640-5p<br>hsa-miR-3690<br>hsa-miR-346<br>hsa-miR-214-5p<br>hsa-miR-296-3p<br>hsa-miR-216a-3p<br>hsa-miR-128-3p<br>hsa-miR-3681-3p<br>hsa-miR-1193<br>hsa-miR-542-3p<br>hsa-miR-1286<br>hsa-miR-3186-3p<br>hsa-miR-149-5p<br>hsa-miR-605-3p<br>hsa-miR-491-5p<br>hsa-miR-3127-5p<br>hsa-miR-3918<br>hsa-miR-339-3p<br>hsa-miR-129-1-3p<br>hsa-miR-129-2-3p<br>hsa-miR-331-3p<br>hsa-miR-650<br>hsa-miR-3612<br>hsa-miR-510-5p<br>hsa-miR-4436a<br>hsa-miR-5000-3p<br>hsa-miR-1197<br>hsa-miR-642b-3p<br>hsa-miR-642a-3p<br>hsa-miR-3173-5p<br>hsa-miR-6799-3p<br>hsa-miR-2114-5p<br>hsa-miR-627-5p<br>hsa-miR-195-5p
Tarbase	hsa-miR-151a-3p<br>hsa-miR-424-5p<br>hsa-miR-16-5p<br>hsa-miR-103a-3p<br>hsa-miR-107<br>hsa-miR-17-3p<br>hsa-miR-22-3p<br>hsa-miR-522-5p<br>hsa-let-7e-5p

	hsa-miR-100-5p<br>hsa-miR-103a-2-5p<br>hsa-miR-1207-5p<br>hsa-miR-1224-5p<br>hsa-miR-128-3p<br>hsa-miR-140-5p<br>hsa-miR-149-3p<br>hsa-miR-15a-5p<br>hsa-miR-15b-5p<br>hsa-miR-192-3p<br>hsa-miR-224-5p<br>hsa-miR-2277-3p<br>hsa-miR-27a-3p<br>hsa-miR-27b-3p<br>hsa-miR-29a-3p<br>hsa-miR-29b-3p<br>hsa-miR-29c-3p<br>hsa-miR-3199<br>hsa-miR-320d<br>hsa-miR-320e<br>hsa-miR-330-3p<br>hsa-miR-339-3p<br>hsa-miR-33b-3p<br>hsa-miR-3605-5p<br>hsa-miR-421<br>hsa-miR-449b-5p<br>hsa-miR-484<br>hsa-miR-486-3p<br>hsa-miR-518c-5p<br>hsa-miR-758-3p<br>hsa-miR-99a-3p<br>hsa-miR-99a-5p<br>hsa-miR-99b-5p<br>kshv-miR-K12-2-3p<br>hsa-miR-155-5p<br>hsa-miR-23b-3p<br>hsa-let-7b-5p<br>hsa-miR-20a-5p
RegRNA	hsa-miR-27b-3p<br>hsa-miR-324-3p<br>hsa-miR-30c-1-3p<br>hsa-miR-30c-2-3p<br>hsa-miR-6084<br>hsa-miR-6810-3p

	hsa-miR-6810-5p<br>hsa-miR-6813-3p<br>hsa-miR-6823-5p<br>hsa-miR-7113-3p<br>hsa-miR-1260a

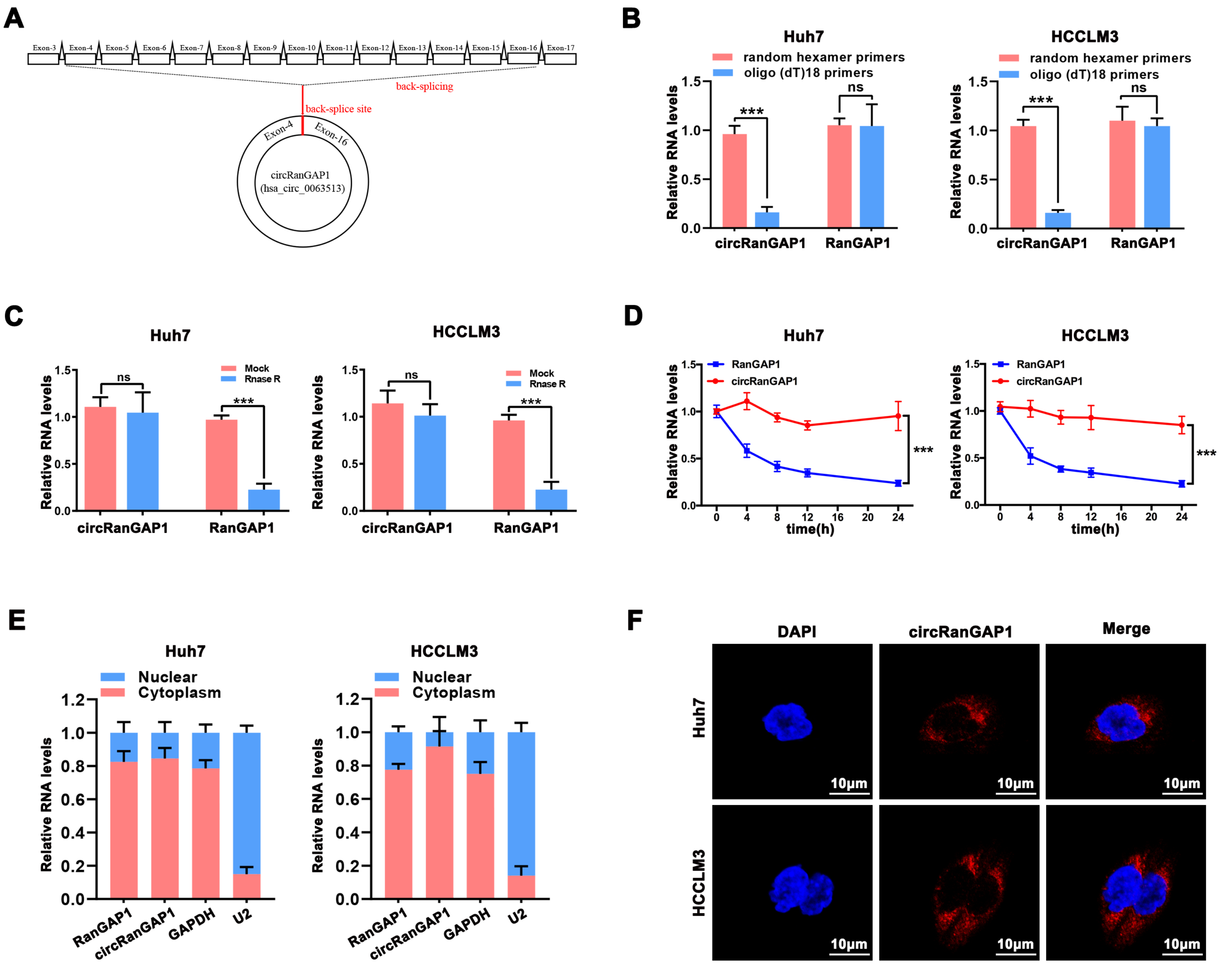

Supplementary Figure 1

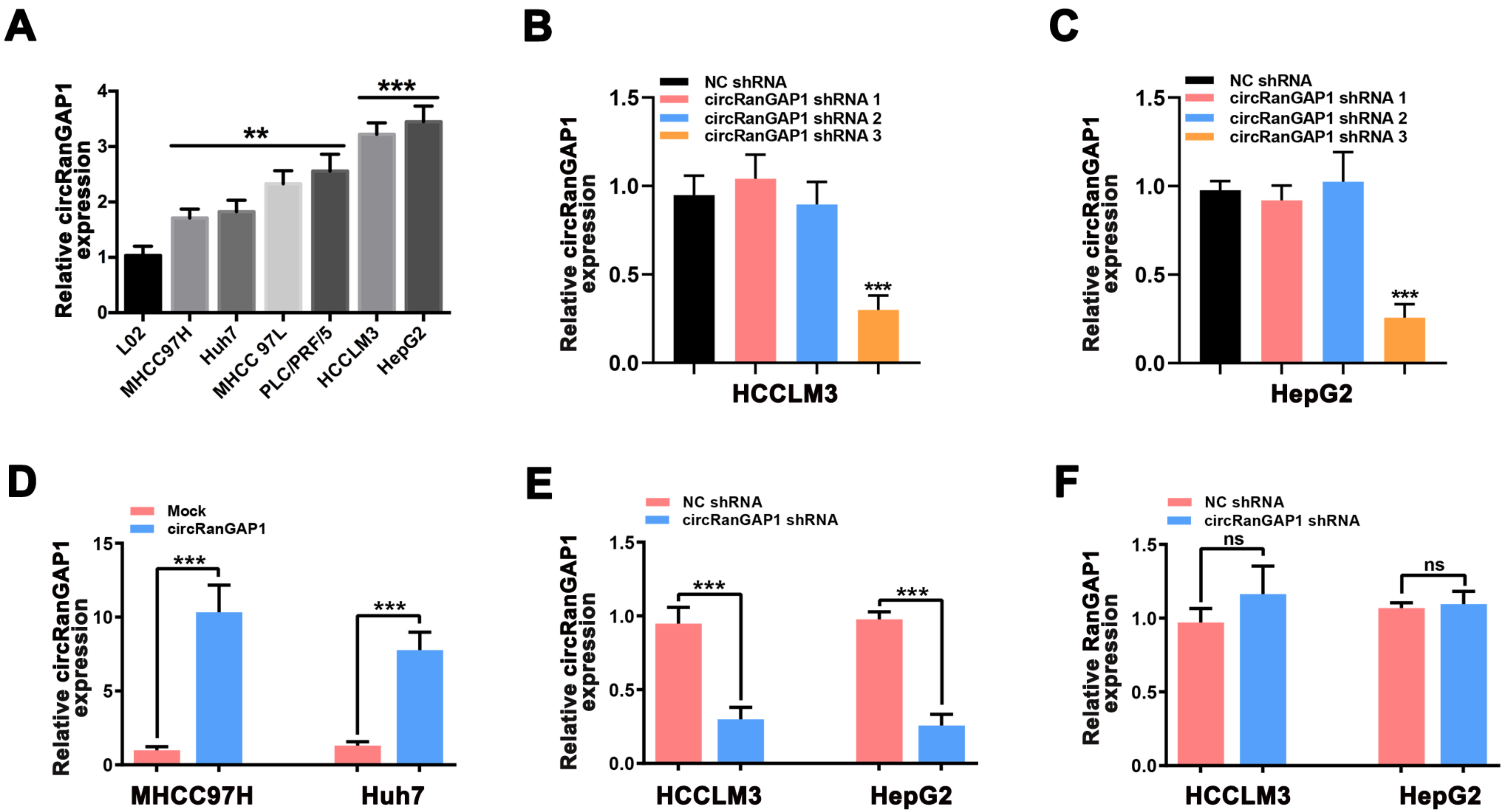

Supplementary Figure 2

A

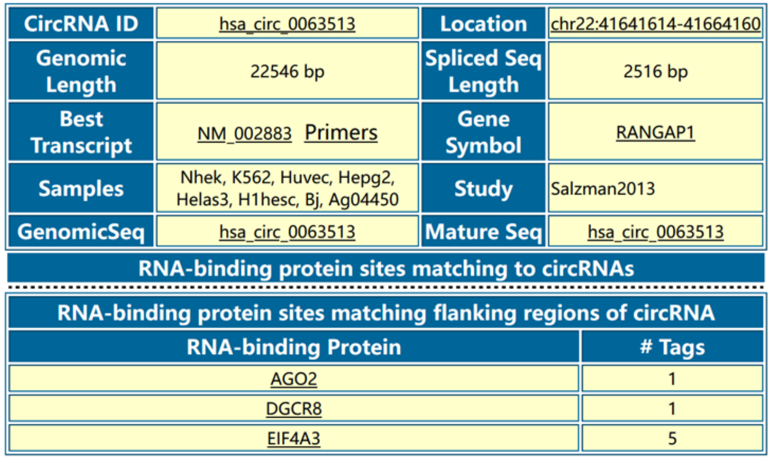

CircRNA ID	hsa_circ_0063513	Location	chr22:41641614-41664160
Genomic Length	22546 bp	Spliced Seq Length	2516 bp
Best Transcript	NM_002883 Primers	Gene Symbol	RANGAP1
Samples	Nhek, K562, Huvec, Hepg2, Helas3, H1hesc, Bj, Ag04450	Study	Salzman2013
GenomicSeq	hsa_circ_0063513	Mature Seq	hsa_circ_0063513

RNA-binding protein sites matching to circRNAs

RNA-binding protein sites matching flanking regions of circRNA

RNA-binding Protein	# Tags
AGO2	1
DGCR8	1
EIF4A3	5

B C

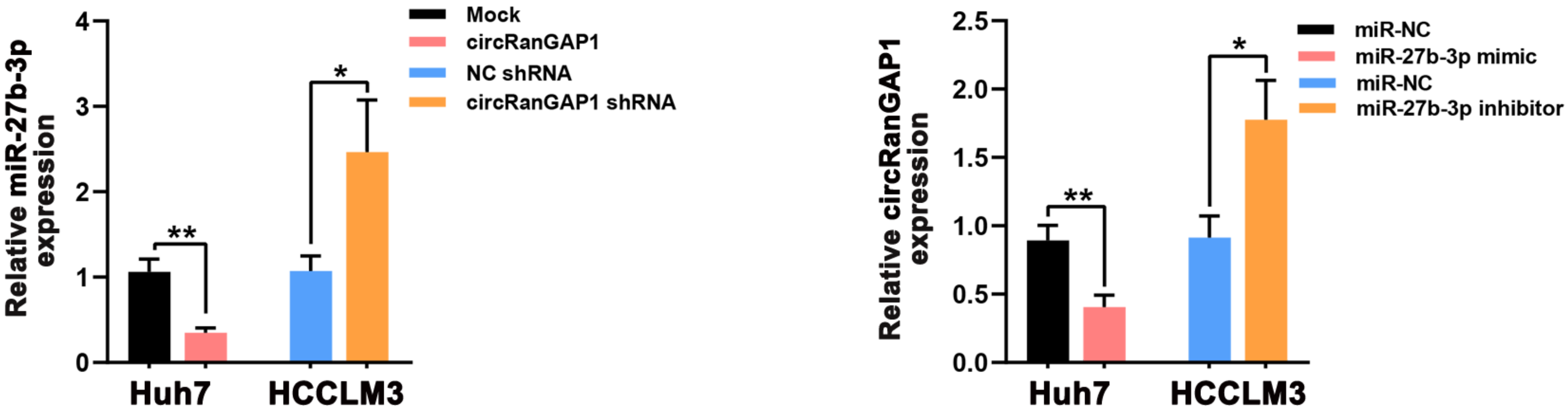

Supplementary Figure 3

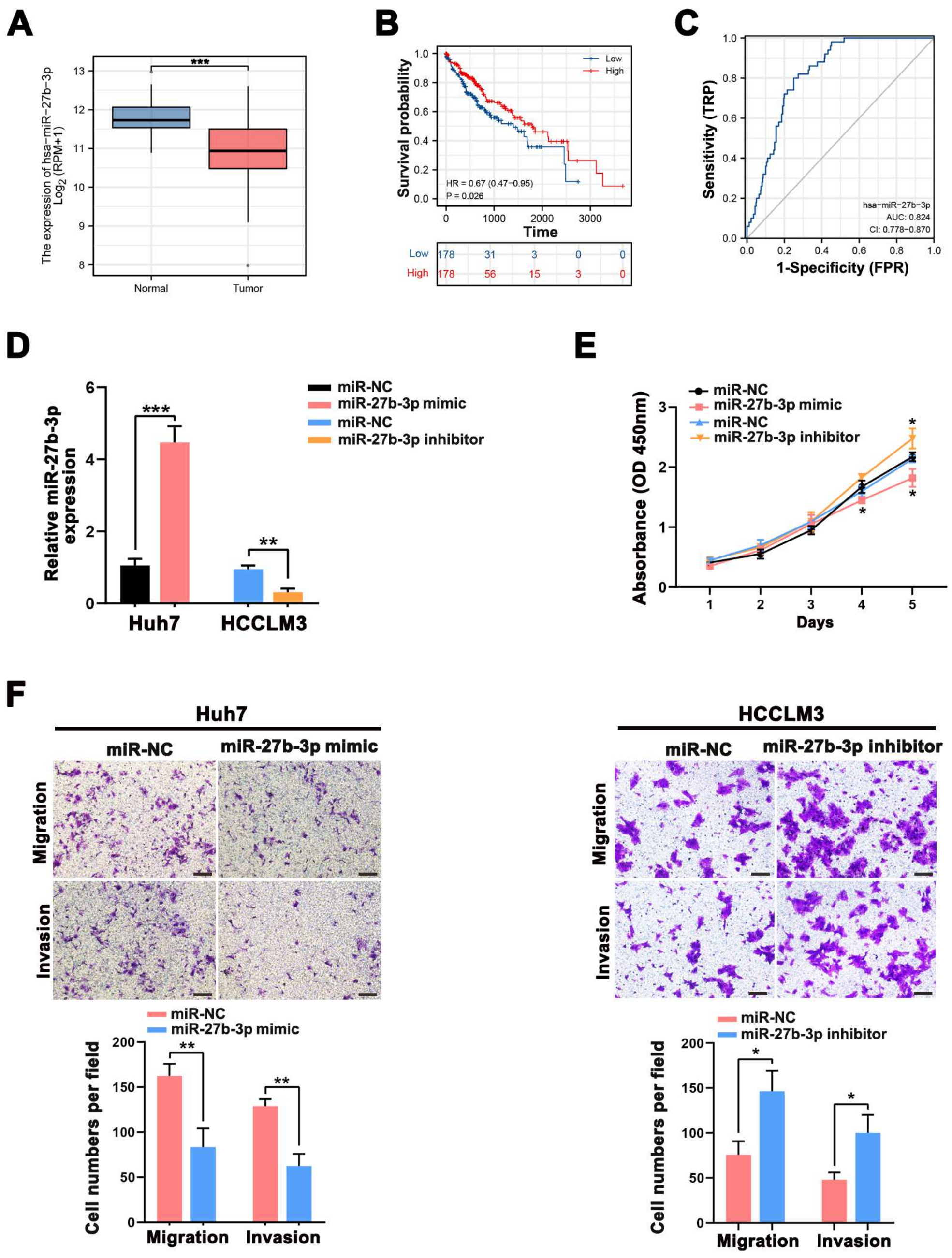

Supplementary Figure 4

A

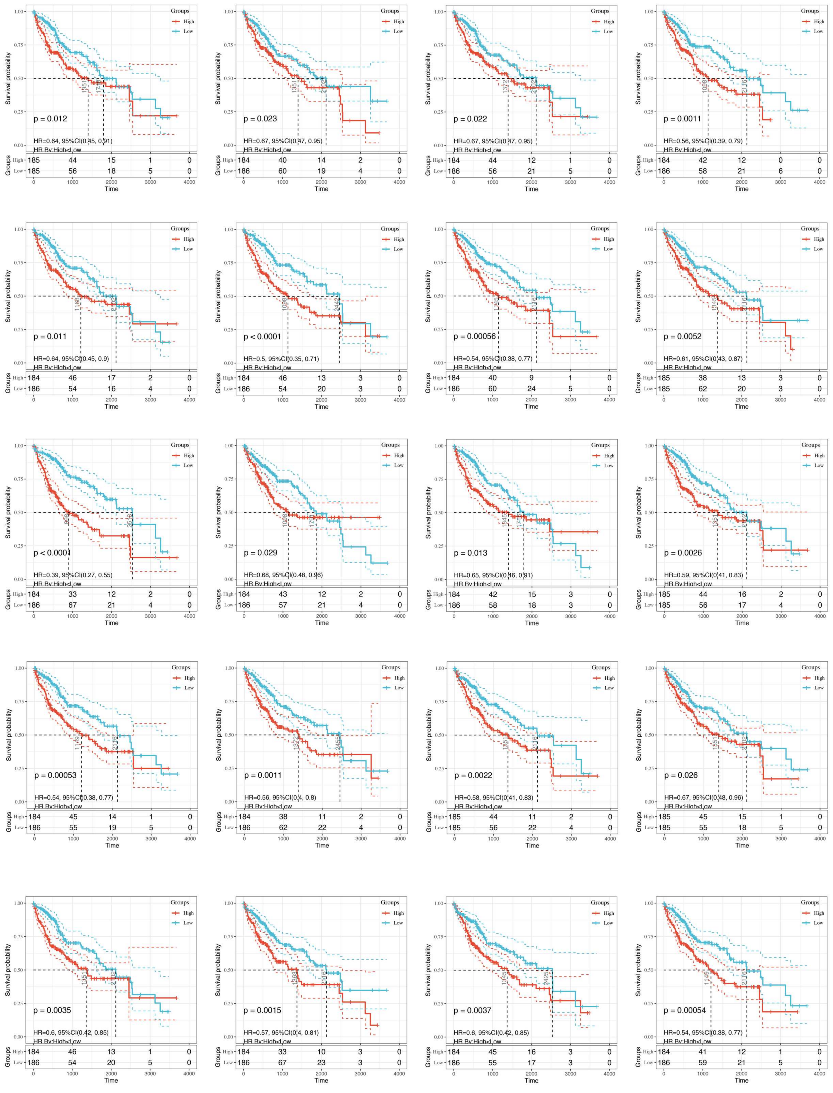

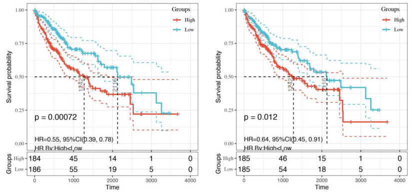

B

Node

target	degree	closeness	betweenness
has-miR-27b-3p	22	22	448
NRAS	4	13	2
CREB1	4	13	2
SOS1	3	12.5	0
PPARG	2	12	0

Supplementary Figure 5

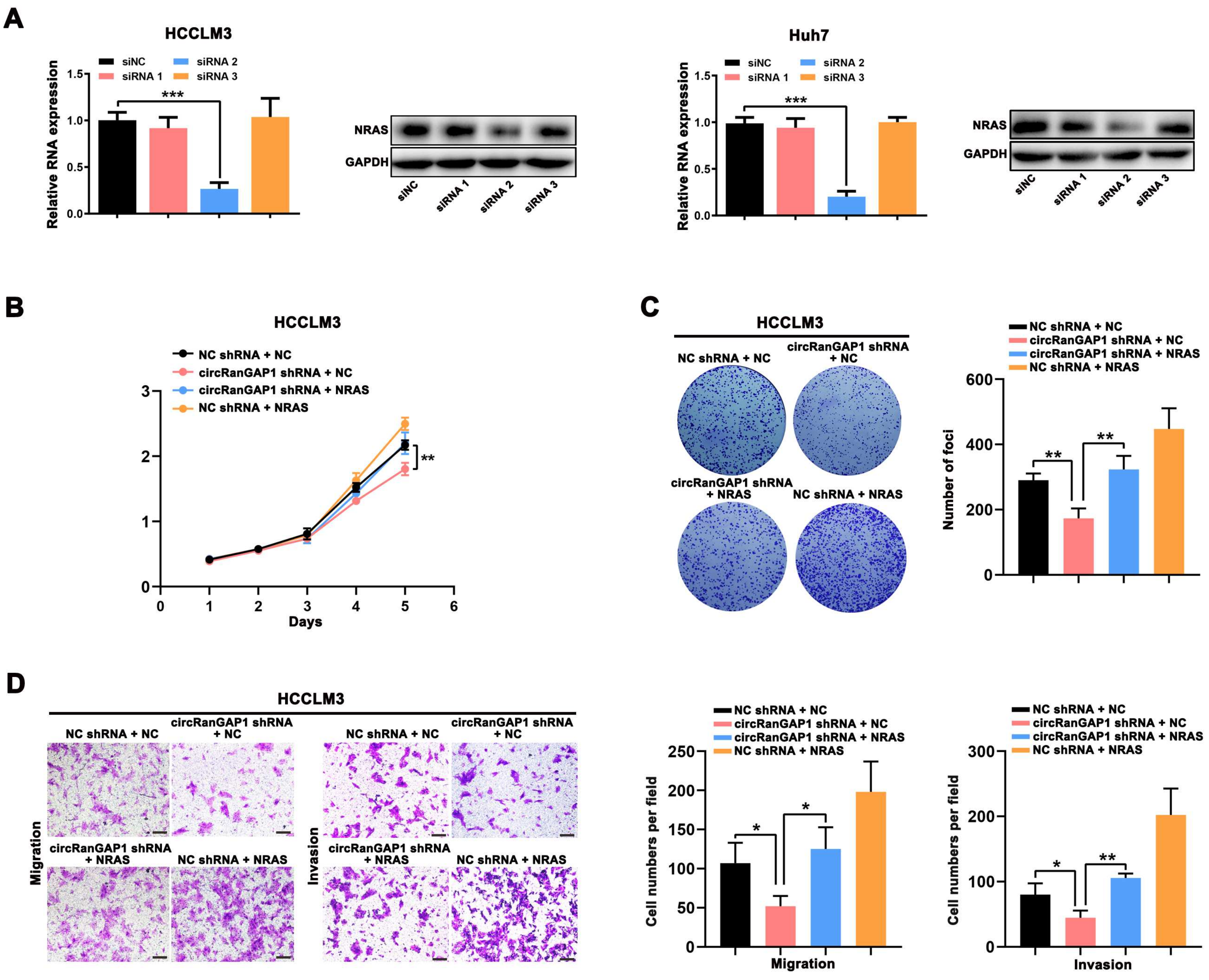

Supplementary Figure 6

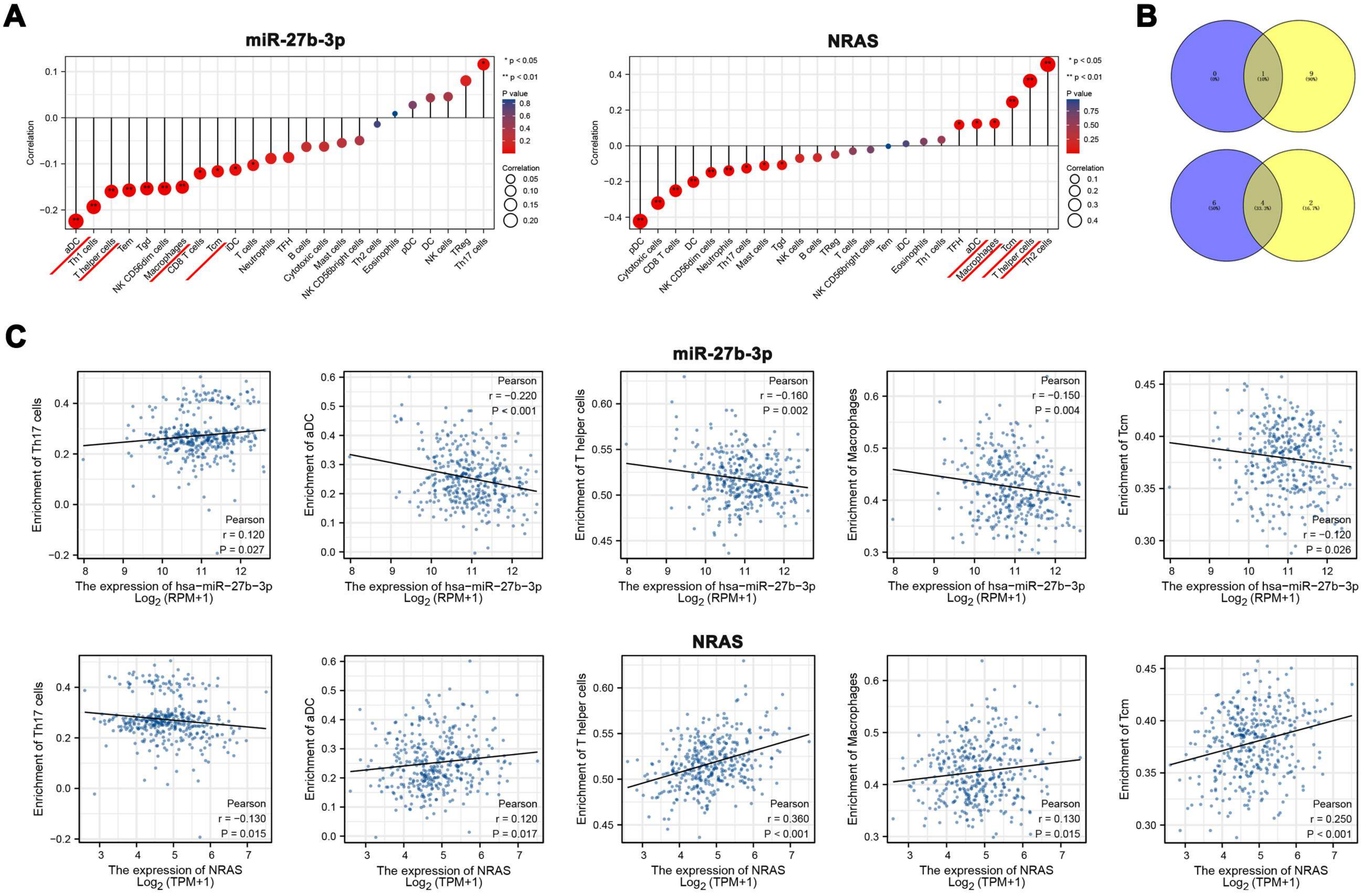

Supplementary Figure 8

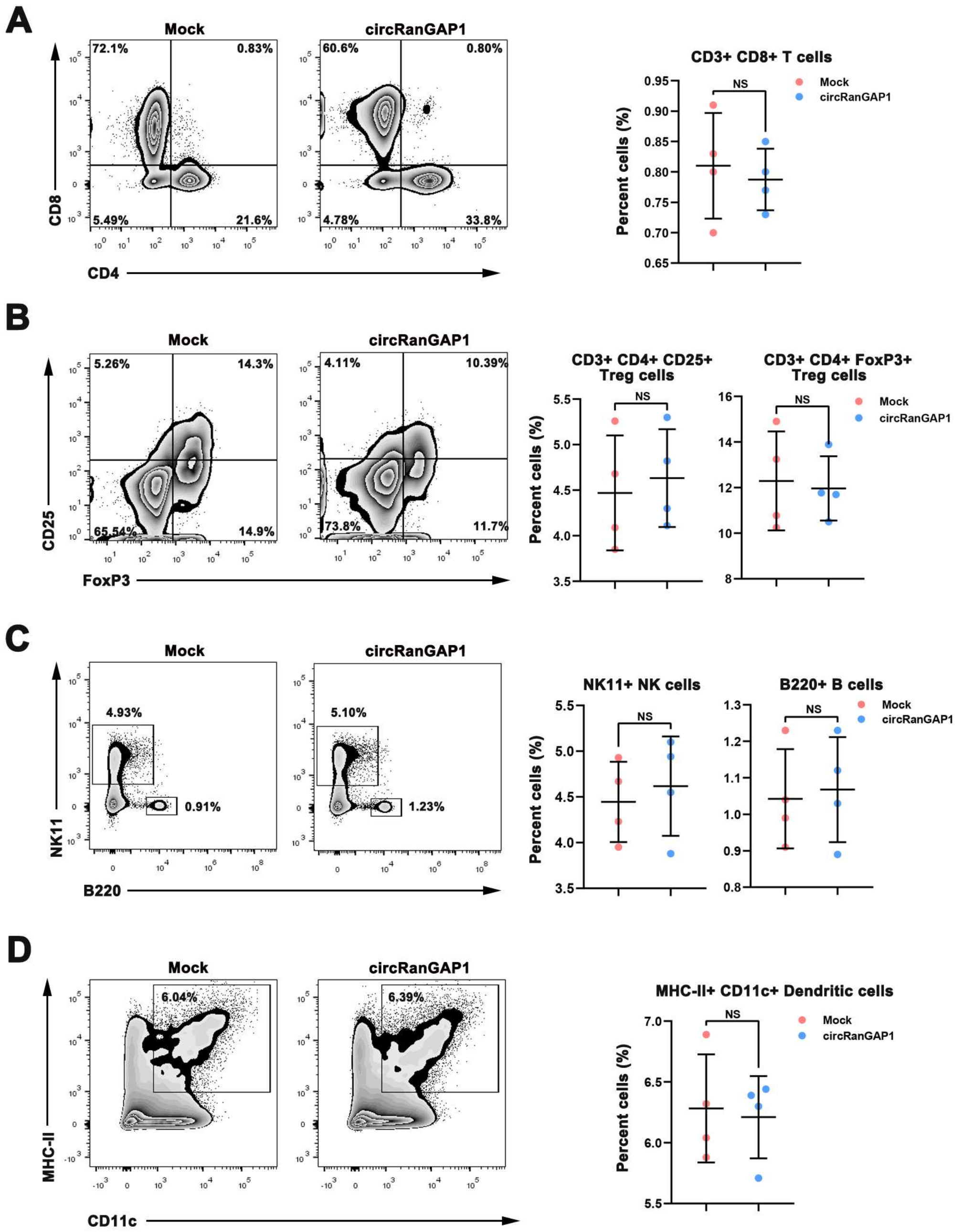

Supplementary Figure 9
